# The influence of soil age on ecosystem structure and function across biomes

**DOI:** 10.1038/s41467-020-18451-3

**Published:** 2020-09-18

**Authors:** Manuel Delgado-Baquerizo, Peter B. Reich, Richard D. Bardgett, David J. Eldridge, Hans Lambers, David A. Wardle, Sasha C. Reed, César Plaza, G. Kenny Png, Sigrid Neuhauser, Asmeret Asefaw Berhe, Stephen C. Hart, Hang-Wei Hu, Ji-Zheng He, Felipe Bastida, Sebastián Abades, Fernando D. Alfaro, Nick A. Cutler, Antonio Gallardo, Laura García-Velázquez, Patrick E. Hayes, Zeng-Yei Hseu, Cecilia A. Pérez, Fernanda Santos, Christina Siebe, Pankaj Trivedi, Benjamin W. Sullivan, Luis Weber-Grullon, Mark A. Williams, Noah Fierer

**Affiliations:** 1 Departamento de Sistemas Físicos, Químicos y Naturales, Universidad Pablo de Olavide, 41013 Sevilla, Spain; 2 Cooperative Institute for Research in Environmental Sciences, University of Colorado, Boulder, CO 80309 USA; 3 Department of Forest Resources, University of Minnesota, St. Paul, MN 55108 USA; 4 Hawkesbury Institute for the Environment, Western Sydney University, Penrith, NSW 2751 Australia; 5 Department of Earth and Environmental Sciences, Michael Smith Building, The University of Manchester, Oxford Road, Manchester, M13 9PT UK; 6 Centre for Ecosystem Studies, School of Biological, Earth and Environmental Sciences, University of New South Wales, Sydney, NSW 2052 Australia; 7 School of Biological Sciences, The University of Western Australia, 35 Stirling Hwy, Crawley (Perth), WA 6009 Australia; 8 Asian School of the Environment, Nanyang Technological University, 50 Nanyang avenue, Singapore, 639798 Singapore; 9 US Geological Survey, Southwest Biological Science Center, Moab, UT USA; 10 Instituto de Ciencias Agrarias, Consejo Superior de Investigaciones Científicas, Serrano 115 bis, 28006 Madrid, Spain; 11 Institute of Microbiology, University of Innsbruck, Technikerstr. 25, Innsbruck, 6020 Austria; 12 Department of Life and Environmental Sciences and Sierra Nevada Research Institute, University of California Merced, Merced, California 95343 USA; 13 Key Laboratory for Humid Subtropical Eco-geographical Processes of the Ministry of Education, School of Geographical Science, Fujian Normal University, 350007 Fuzhou, China; 14 Faculty of Veterinary and Agricultural Sciences, The University of Melbourne, Parkville, VIC 3010 Australia; 15 CEBAS-CSIC. Department of Soil and Water Conservation. Campus Universitario de Espinardo, 30100 Murcia, Spain; 16 GEMA Center for Genomics, Ecology & Environment, Faculty of Interdisciplinary Studies, Universidad Mayor, Camino La Pirámide, 5750 Huechuraba Santiago, Chile; 17 Instituto de Ecología y Biodiversidad, Las Palmeras, 3425 Santiago, Chile; 18 School of Geography, Politics and Sociology, Newcastle University, Newcastle, UK; 19 Centre for Microscopy, Characterization and Analysis, The University of Western Australia, Perth, WA 6009 Australia; 20 Crop, Livestock and Environment Division, Japan International Research Centre for Agricultural Sciences, Tsukuba Ibaraki, 305-8656 Japan; 21 Department of Agricultural Chemistry, National Taiwan University, Taipei, 10617 Taiwan; 22 Instituto de Geología, Universidad Nacional Autónoma de México, Ciudad Universitaria, México, D.F. CP 04510 Mexico; 23 Microbiome Network and Department of Agricultural Biology, Colorado State University, Fort Collins, 80523 CO USA; 24 Department of Natural Resources and Environmental Science, University of Nevada, Reno, NV 89557 USA; 25 Global Drylands Center, Arizona State University, Tempe, AZ USA; 26 School of Life Sciences, Arizona State University, Tempe, AZ USA; 27 School of Sustainability, Arizona State University, Tempe, AZ USA; 28 School of Plant and Environmental Sciences, Virginia Polytechnic Institute and State University, Blacksburg, VA USA; 29 Department of Ecology and Evolutionary Biology, University of Colorado, Boulder, CO 80309 USA

**Keywords:** Biogeochemistry, Ecosystem ecology, Ecosystem services, Macroecology

## Abstract

The importance of soil age as an ecosystem driver across biomes remains largely unresolved. By combining a cross-biome global field survey, including data for 32 soil, plant, and microbial properties in 16 soil chronosequences, with a global meta-analysis, we show that soil age is a significant ecosystem driver, but only accounts for a relatively small proportion of the cross-biome variation in multiple ecosystem properties. Parent material, climate, vegetation and topography predict, collectively, 24 times more variation in ecosystem properties than soil age alone. Soil age is an important local-scale ecosystem driver; however, environmental context, rather than soil age, determines the rates and trajectories of ecosystem development in structure and function across biomes. Our work provides insights into the natural history of terrestrial ecosystems. We propose that, regardless of soil age, changes in the environmental context, such as those associated with global climatic and land-use changes, will have important long-term impacts on the structure and function of terrestrial ecosystems across biomes.

## Abstract (web summary)

Soil age is thought to be an important driver of ecosystem development. Here, the authors perform a global survey of soil chronosequences and meta-analysis to show that, contrary to expectations, soil age is a relatively minor ecosystem driver at the biome scale once other drivers such as parent material, climate, and vegetation type are accounted for.

## Introduction

Terrestrial ecosystem development^1–5^, which involves changes in ecosystem structure and function over time scales of centuries to millennia, is widely thought to be controlled by the five state factors that also control pedogenesis: time of development (i.e., age), climate, topography, parent material, and organisms (notably vegetation)^1–3^. Current hypotheses propose that soil age (i.e., substrate age as a proxy for extent of soil formation and weathering) is a major ecosystem driver at a local scale^6–13^. For instance, soils take from hundreds to millions of years to develop within a given ecosystem, resulting in important changes in carbon (C) stocks, C:nitrogen (N):phosphorus (P) ratios, and soil pH^1–5^. However, soil age is not the only driver of terrestrial ecosystem structure and function; otherwise, all ecosystems with the same soil age would share the same chemical and physical characteristics. An assessment of the relative importance of soil age compared with the other major state factors that can operate at larger spatial scales (i.e., parent material, climate, vegetation type, and topography), however, has not been attempted. In fact, studies quantifying the relative contribution of the five state factors^1–3^ to terrestrial ecosystem development across global biomes have been particularly lacking. One reason for this is that most studies have focused separately on how spatial gradients (i.e., natural environmental variation in climate and vegetation) or temporal gradients (i.e., soil age) influence ecosystem structure and function (but see refs. ^5,13^ at the regional scale). Nevertheless, elucidating the relative importance of these state factors in regulating ecosystem structure and function from local to global scales is a fundamental question in ecology and biogeochemistry^1–3^. Such knowledge would advance our understanding of how ecosystems develop through time, and improve forecasts and management options for ecosystems on a planet subjected to large and interacting changes in climate and land-use.

Over the past few decades, several studies have used long-term soil chronosequences to quantify how local ecosystem development affects key above- and belowground ecosystem properties, biogeochemical cycling, and the community structure of plants, microbes, and animals over time scales of centuries to millennia^6–13^. However, much less is known about the role of environmental context in driving the absolute values, trajectories, and rates of development over time for multiple structural and functional ecosystem properties across contrasting climates, biomes, and parent material types. A recent regional study^13^ suggests that drier environments fail to show the same strong trends in ecosystem development reported for more mesic ecosystems^4,5,10,12^. However, we still lack a unified understanding of the role of environmental context in driving terrestrial ecosystem development across contrasting global biomes. Herein, we quantify the contribution of soil age relative to other key state factors of ecosystem development (parent material, climate, vegetation type, and topography)^1–3^ in controlling changes in multiple structural and functional ecosystem properties across biomes. Moreover, we investigate changes in multiple ecosystem structural and functional properties during ecosystem development across contrasting ecosystem types.

**Fig. 1:**
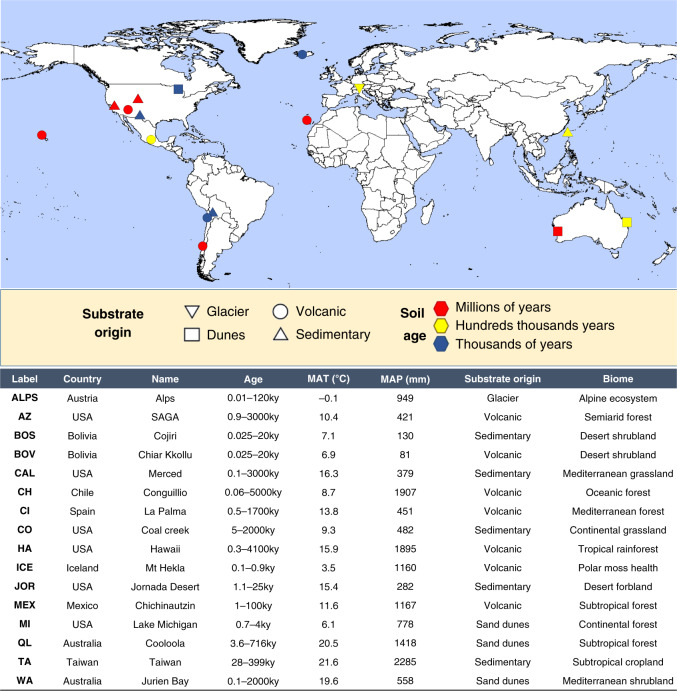
Information on the investigated 16 soil chronosequences. Location, soil age, climate, vegetation, and chronosequence origin for the 16 globally distributed chronosequences included in this study. Blue, yellow, and red locations include chronosequences for soil ages ranging from centuries  to thousands, to hundreds of thousands, and to millions of years, respectively. See Supplementary Table 1 for further details on these chronosequences.

We combine two complementary approaches to address the above-mentioned knowledge gaps. First, we collect an extensive amount of new field data from 16 chronosequences^14^ across contrasting biomes from six continents (Fig. 1), and obtain information for 32 topsoil, plant, and microbial ecosystem properties (Supplementary Table 3). We focus on topsoil (0–10 cm soil depth) for three reasons. First, this is the most commonly used soil sampling depth in comparable studies. Second, this uppermost layer is typically biologically the most active in terms of plant roots, microbial biomass, labile nutrient pools, and C exchange with the atmosphere. Finally, many sites have very shallow soils, making a collection of soils from greater depths impossible. Sampling from the same relative position in the topsoil profile allows us to directly compare soils at the same depth across all chronosequences, and avoid introducing biases associated with differences in soil depth. The 16 chronosequences range from hundreds to millions of years and cover a wide variety of globally distributed vegetation types (including grasslands, shrublands, forests, and croplands), chronosequence origins (volcanic, sedimentary, dunes and glacier) and climates (tropical, temperate, continental, Mediterranean, polar and arid) (Fig. 1 and Supplementary Table 1). For example, mean annual temperature, precipitation, and elevation range from −2.8 to 21.7 °C, 81 to 2347 mm, and 4 to 3716 m, respectively. Importantly, the sampled soil chronosequences^14^ include soil age gradients of comparable length (age) for mesic/warm and dry/cold ecosystems, and across contrasting vegetation types and edaphic conditions (e.g., volcanic vs. sedimentary parent material) (Fig. 1 and Supplementary Table 1). This design allows us to disentangle the relative contribution of soil age, climate, vegetation, parent materials, and topography in driving the structure and function of terrestrial ecosystems across biomes.

We then undertake a meta-analysis of existing chronosequence data from the literature. This adds 48 comparable globally distributed chronosequences to our analysis and captures a wider spectrum of conditions (Supplementary Table 2 and Supplementary Methods 1 and 2). While some locations are notably absent (e.g., continental Africa), and should be the focus of future research, our two research approaches include chronosequences from six continents ranging in soil age from hundreds to millions of years. These additional soil chronosequences also encompass a wide range of climatic conditions (e.g., from tropical forests to deserts), major vegetation types (grasslands, shrublands and coniferous and angiosperm forests) and parent material types (e.g., volcanic, sedimentary, and sand dunes; Supplementary Table 2 and Supplementary Methods 1 and 2).

**Fig. 2:**
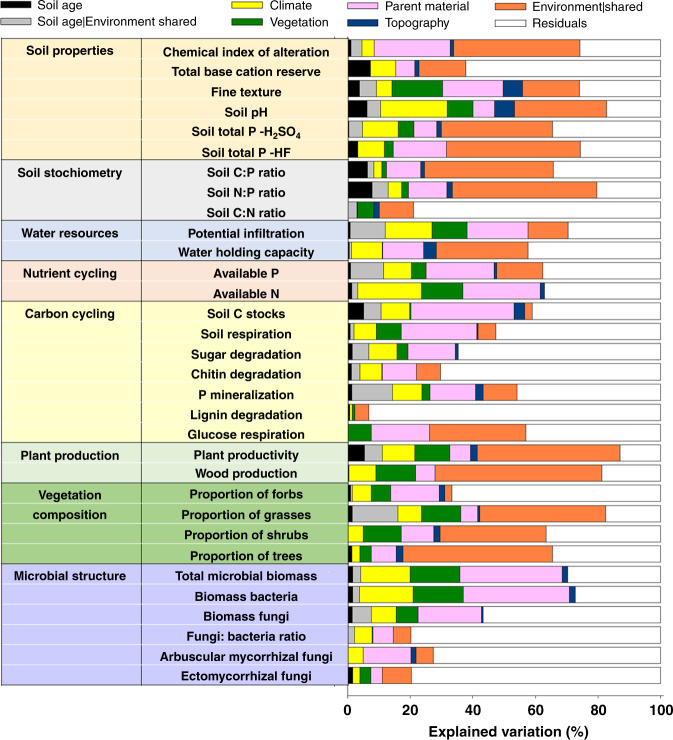
Contribution of the ecosystem development state factors to the structure and function of terrestrial ecosystems across global biomes. Variation Partitioning Modeling was used to evaluate the unique and shared portions of variation in ecosystem properties explained by soil age, climate, vegetation type, parent material, and topography. Environmental | shared refers to the percent of shared variation in ecosystem properties explained by parent material, climate, vegetation type, and topography. Soil age | shared refers to the percent of the shared variation in ecosystem properties explained by soil age, parent material, climate, vegetation type, and topography. *P*-values associated with the unique portions explained by different groups of predictors are available in Supplementary Table 5. Detailed information on the *n*, units, rationale, descriptions, and acronyms for these functional and structural properties can be found in Supplementary Table 3. The CIA (chemical index of alteration) and the TBR (total base cation reserves) indices provide information on the relative extent of weathering.

**Fig. 3:**
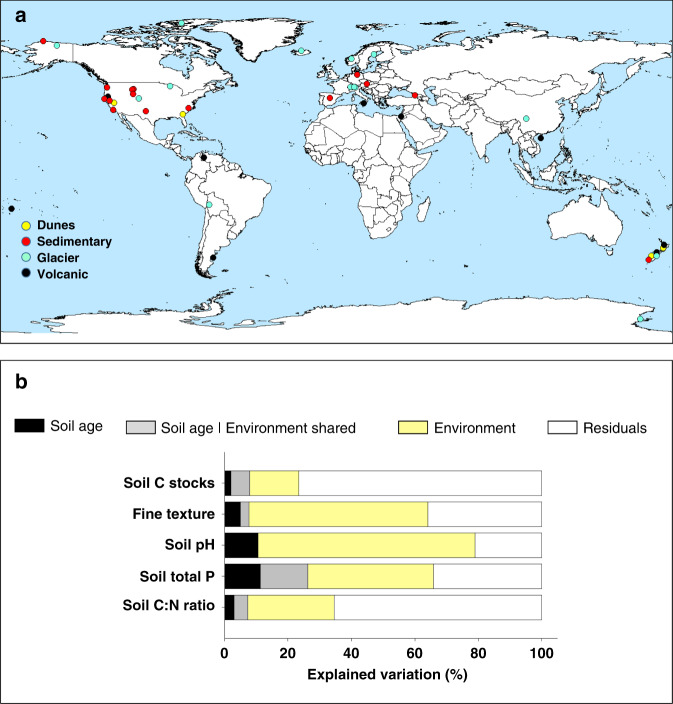
Meta-analysis of 48 soil chronosequences across the globe. **a** Includes the locations for 48 soil chronosequences. Yellow circles = dunes; red circles = sedimentary; blue circles = glacier; black circles = volcanic. **b** Includes Variation Partitioning Modeling evaluating the portion of variation in ecosystem properties explained by (1) soil age alone, (2) soil age and other state factors combined, and (3) other state factors combined. State factor combined refers to the portion of unique and shared variation in ecosystem properties explained by parent material, climate, vegetation type, and topography together. Soil age | shared refers to the percent of the shared variation in ecosystem properties explained by soil age together with parent material, climate, vegetation type, and topography. *n* as follows: soil C:N ratio = 357, soil P = 228, soil pH = 216, texture = 454, soil C stocks = 252. More information on these soil chronosequences can be found in Supplementary Table 2 and Supplementary Methods 1 and 2.

**Fig. 4:**
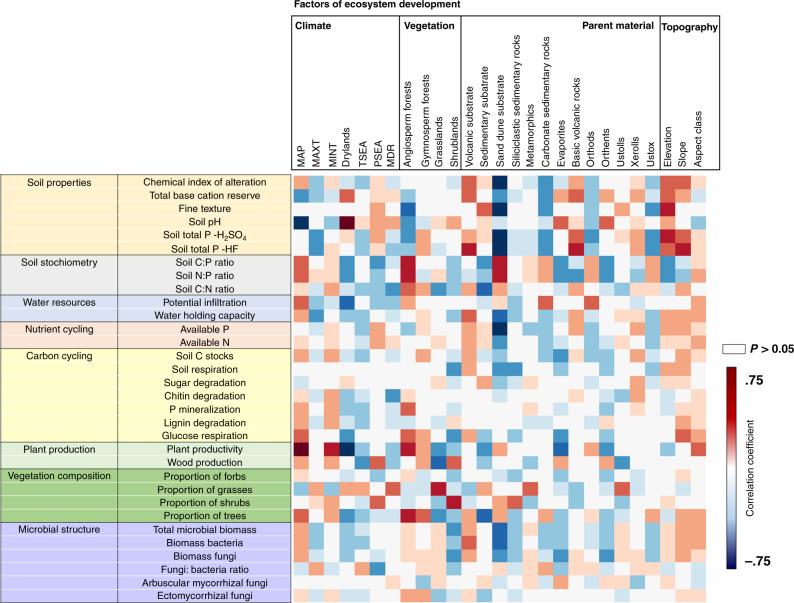
Correlations between environmental factors and ecosystem properties. Partial correlations (Spearman) (see Supplementary Table 3 for the number of samples) between climate, vegetation type, parent material, and topography with ecosystem structure, and functions controlled for soil age and geographical location. See Supplementary Table 3 for a list of functional and structural properties and a rationale for their importance for terrestrial ecosystems. See Supplementary Table 4 for a list of ecosystem development factors and a rationale for their importance for terrestrial ecosystems.

**Fig. 5:**
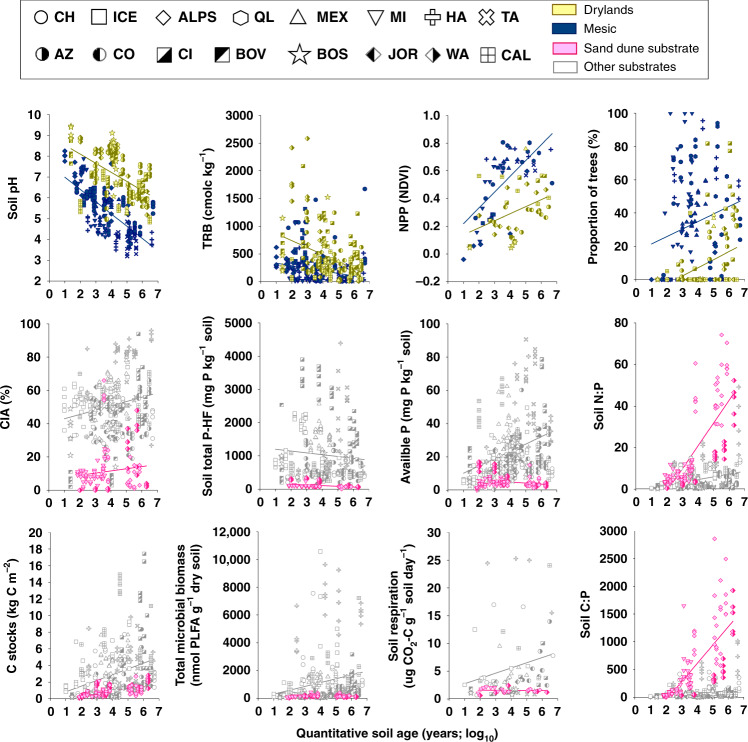
Environmental context determines the rates and trajectories of ecosystem development. Selected relationships between quantitative soil age and ecosystem properties across major biome categories (drylands vs. mesic) and substrate types. See Supplementary Table 6 for statistical details.

**Fig. 6:**
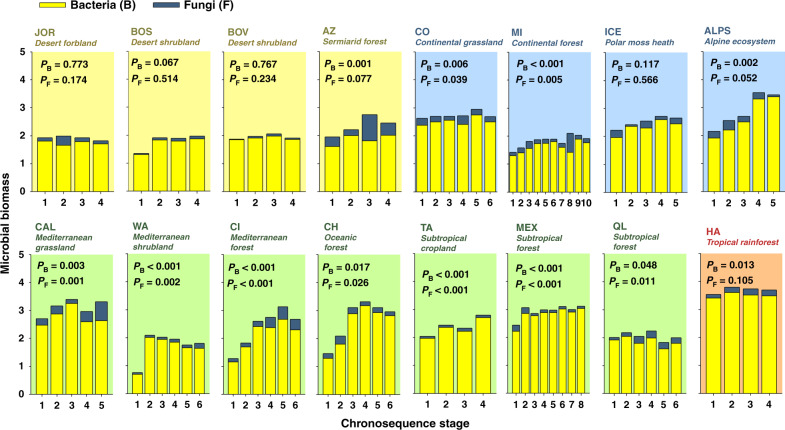
Changes in bacterial and fungal biomass during ecosystem development. Microbial biomass (mean values) = nmol PLFA g^−1^ dry soil (log_10_-transformed). Acronyms for different chronosequences are available in Supplementary Fig. 1. PERMANOVA *P*-values are shown. Chronosequence stage was included as a fixed factor in these analyses (*n* = 5).

**Fig. 7:**
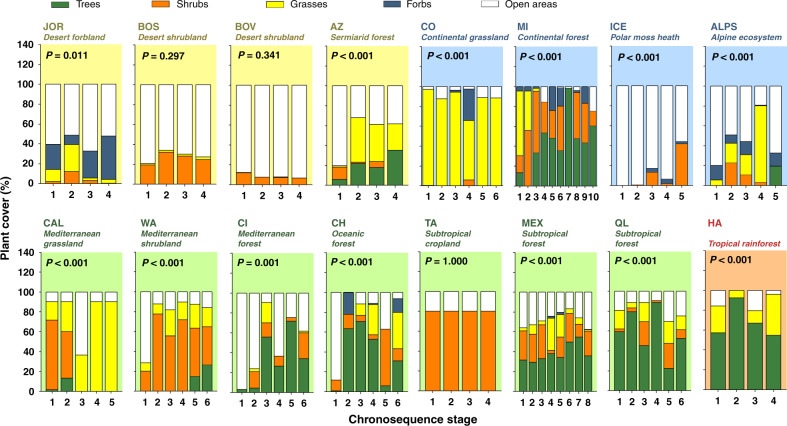
Changes in plant community structure during ecosystem development. Plant cover (mean values) = %. Acronyms for different chronosequences are available in Supplementary Fig. 1. PERMANOVA *P*-values are shown. Chronosequence stage was included as a fixed factor in these analyses (*n* = 3 transects).

**Fig. 8:**
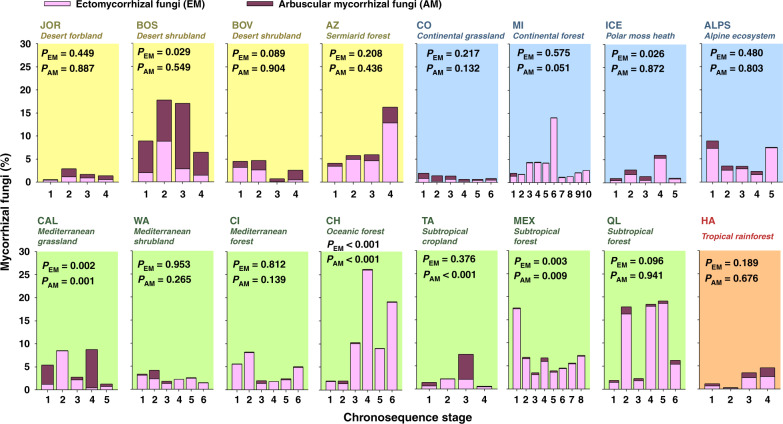
Changes in mycorrhizal community structure during ecosystem development. Mycorrhizal fungi (mean values) = %. PERMANOVA *P*-values are shown. Chronosequence stage was included as a fixed factor in these analyses (*n* = 5).

**Fig. 9:**
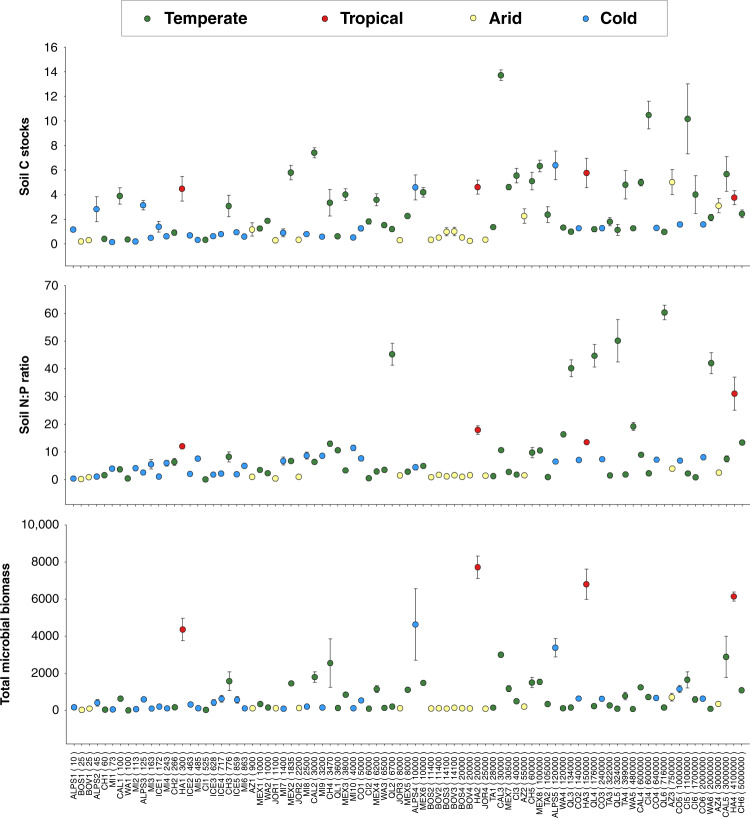
Changes in selected ecosystem properties across climates and time. Changes in soil C stocks, N:P ratios, and total microbial biomass (mean ± SE) across soil ages and biomes (*n* = 5 replicates per chronosequence stage). C stocks in kg C m^−2^; microbial biomass in nmol PLFA g^−1^ dry soil. Soil age (years) in brackets. Green circle = temperate; yellow circle = arid; blue circle = cold (continental and polar); red circle = tropical.

**Fig. 10:**
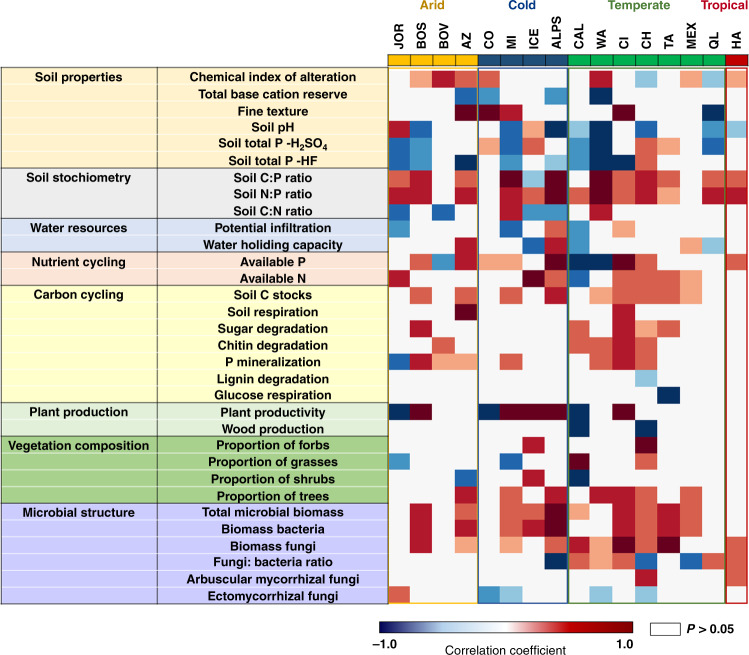
Changes in the structure and function of terrestrial ecosystems during ecosystem development. Correlations (Spearman) among chronosequence stage and ecosystem properties. See Fig. 1 for the location and acronyms of these chronosequences. Statistically non-significant correlations (*P* > 0.05) are shown in white. Numeric values and *n* for all correlations are in Supplementary Table 6. Detailed information on the *n*, units, rationale, descriptions, and acronyms for these functional and structural properties are in Supplementary Table 3. The chemical index of alteration (CIA) and the total base cation reserves (TBR) indices provide information on the relative extent of weathering.

We show that soil age is a significant, but relatively weak, ecosystem driver across biomes, and provide evidence that, on average, parent material, climate, vegetation, and topography together explain 24 times more variation in multiple ecosystem properties than soil age alone (Figs. 1–3). Moreover, our work indicates that the environmental context determines the rates and trajectories of ecosystem development in multiple ecosystem properties across biomes (Figs. 4–9). Here soils that developed on sandy substrates always show lower levels, and slower over time development, of soil microbial biomass, C stocks, P availability, and N:P and C:P ratios, than other substrates (e.g., volcanic). Moreover, irrespective of soil age, drier ecosystems tend to have more alkaline soils and less plant productivity development over time, than more mesic ecosystems. That said, soil age is not insignificant (Figs. 2 and 10). Soil age could help the fine-tuning of global ecosystem models and is an important ecosystem driver at the local scale—as supported by some consistent patterns in the changes of ecosystem properties over time (Fig. 10). This appears particularly important for slow-changing ecosystem properties associated with a biological activity such as soil N:P and C:P ratios, bacterial and fungal biomass, and C stocks, which consistently increase with soil age in more than half of the studied chronosequences (Fig. 10). Taken together, our findings provide key insights into the natural history of terrestrial ecosystems, and suggest that global climatic and land-use changes could have implications for the longer-term trajectories in ecosystem development.

## Results

### Importance of soil age as an ecosystem driver across biomes

Across biomes, we found that soil age was a significant but relatively weak ecosystem driver of change. Taken together, parent material, climate, vegetation type, and topography predicted an average of 24 times more variation in multiple ecosystem properties than soil age alone (Fig. 2 and Supplementary Tables 3–5). Further, soil age (across 32 ecosystem properties), explained a relatively small portion of unique (2.1%) and shared (with environment; 3.5%) variation across biomes (Fig. 2 and Supplementary Tables 3–5). More specifically, our results indicate that spatial state factors explained, on average, seven (parent material alone; 14.5%), four (climate alone; 8.0%), and three (vegetation type alone; 6.1%) times more variation in ecosystem properties than soil age alone (2.1%), for the 32 measured soil, plant and microbial ecosystem properties (Fig. 2 and Supplementary Tables 3–5). Parent material, climate, and vegetation were particularly good predictors of bacterial biomass (33.9%), soil pH (21.2%), and texture (16.2%), respectively. Although of lesser importance, soil age still predicted a unique and significant portion of the variation for more than two-thirds of the evaluated ecosystem properties across biomes, and, on average, soil age explained a slightly higher proportion of variation in ecosystem properties to that explained by topography alone (1.5%; Fig. 2 and Supplementary Table 5). Remarkably, soil age-predicted important unique fractions of the variation in soil N:P (7.8%) and C:P ratios (6.3%), soil pH (6.3%), and total base cation reserve (TBR) index (a surrogate of soil weathering, Supplementary Table 4; 7.3%), plant productivity (5.4%) and C stocks (5.1%) across sites (Fig. 2; Supplementary Table 5). Supporting our main findings, our meta-analysis of published data, based on 48 additional soil chronosequences, also revealed that soil age explained a relatively smaller, but significant and unique, portion of variation compared with that predicted collectively by the remaining state factors (Fig. 3).

We then used regression analyses and partial Spearman correlations to investigate the associations between environmental factors and ecosystem properties. Spearman rank correlations measure the direction and strength of association between two ranked variables, statistically controlling for soil age, and do not require normality of data. Our analyses provided further evidence that environmental context, rather than soil age, determine the structure and function of terrestrial ecosystems across biomes (Figs. 4–9 and Supplementary Figs. 1–4). Drier and sandy ecosystems (Fig. 4) had lower absolute values for ecosystem structure and function than wetter soils developed over other substrates, such as those from volcanic soils (Fig. 4). For example, soils developed over sandy substrates tended to have lower measures of total and available soil P, C stocks, soil respiration rates, microbial biomass, and chemical index of alteration (i.e., CIA; higher levels of weathering) than soils developed over other substrate types (e.g., sedimentary or volcanic; Figs. 4, 5 and Supplementary Table 6). Sandy soils also showed steeper soil N:P and C:P increases with time compared with soils from other substrates, and supported overall flat or negative developments in soil respiration, microbial biomass, C stocks, and available P over time, compared with other substrates (Fig. 5). Moreover, irrespective of soil age, drier and non-forested ecosystems tend to have lower measures of soil C stocks, C:P and N:P ratios, plant productivity, the proportion of trees, and microbial biomass, compared with more mesic ecosystems (Figs. 4–8 and Supplementary Table 6). Furthermore, mesic and forest ecosystems always had more acidic soils, a greater chemical index of alteration (i.e., CIA; higher levels of weathering), a larger proportion of ectomycorrhizal fungi than did drier environments, and steeper increases in plant productivity with time (Figs. 4–8 and Supplementary Table 6 and Supplementary Figs. 1–4).

The importance of environmental context in controlling ecosystem structure and function is also apparent when directly comparing soils with the same age, but from contrasting environmental settings. For example, 1000-year-old soils from temperate Mexican forests (MEX) and Australian shrublands (WA) have two times more C and microbial biomass, and four times higher N:P ratios, than soils of the same age from arid ecosystems in Arizona (AZ) and New Mexico (JOR; Fig. 9). Similarly, a 20,000-year-old soil from a tropical forest in Hawaii (HA) has, on average, 13, 71, and 13 times more C, N:P ratio and total microbial biomass, respectively, than soils from arid ecosystems in New Mexico (JOR) and Bolivia (BOS and BOV; Fig. 9) of similar age. Finally, 3–4 million-year-old soils from volcanic forests in Hawaii (HA) have 17 times more microbial biomass and 12 times higher N:P ratios than similar types of soils from Arizona (AZ; Fig. 9).

### Changes in ecosystem properties within soil chronosequences

We then explored the development of terrestrial structure and function within ecosystems, and show that soil age is an important local-scale ecosystem driver. We found consistent patterns in how ecosystem properties change during ecosystem development across biomes for over two-thirds of all the evaluated ecosystem properties. These consistent patterns included overall positive, negative, or neutral changes with soil age (Fig. 10). For example, we identified general positive trends associated with soil age for nine of the 32 ecosystem properties (Figs. 4–8 and 10). These include general increases in soil N:P (13 of 16 ecosystems) and C:P (11 of 16 ecosystems) ratios as soil develops (Fig. 10 and Supplementary Fig. S4). Moreover, we found increases in total microbial (10 of 16 ecosystems), bacterial (9 of 16 ecosystems) and fungal (9 of 16 ecosystems) biomass, C stocks (9 of 16 ecosystems), soil available P (8 of 16 ecosystems), tree cover (7 of 16 ecosystems), CIA (7 of 16 ecosystems), and P mineralization (7 of 16 ecosystems) with soil age (Figs. 4–8, 10; Supplementary Fig. 3). Similarly, we found overall declines over time for soil total P (P extracted with hydrofluoric acid, P-HF) and soil pH (eight of 16 ecosystems; Figs. 8 and 10 and Supplementary Fig. 1). Additionally, we found a consistent general lack of changes in lignin degradation (15 of 16 ecosystems), glucose respiration (15 of 16 ecosystems), soil respiration (14 of 16 ecosystems), % of wood resources (14 of 16 ecosystems) and percent of arbuscular mycorrhizal fungi (14 of 16 ecosystems), percent of grasses, shrubs, and forbs (>12 of 16 ecosystems), and potential infiltration (12 of 16 ecosystems) (Figs. 4–6 and 10). Finally, we found inconsistent patterns in the changes of other ecosystem properties during ecosystem development and across biomes. For example, sugar and chitin degradation potential generally increased with soil age in temperate ecosystems (four of seven ecosystems), but not in other biomes (Fig. 10). Fungal:bacterial ratios tended to increase with soil age in temperate and tropical ecosystems (five of eight ecosystems), but not in cold and arid environments (Figs. 4 and 10). Plant productivity tended to increase with soil age in cold ecosystems (three of four ecosystems). Lastly, soil C:N ratio tended to decrease in cold and arid (four of eight ecosystems), but not in temperate or tropical ecosystems (Fig. 10).

## Discussion

Current hypotheses propose that soil age is a central driver of ecosystem structure and function at local scales^6–13^. By combining an extensive amount of new cross-biome field data across 16 soil chronosequences distributed along large gradients of climate, vegetation and edaphic conditions, with a global synthesis of existing comparable chronosequence data from the literature, we show that soil age can only explain a relatively small proportion of variation in the changes in ecosystem structure and function across biomes. In particular, we found that, collectively, parent material, climate, vegetation, and topography are more than 24 times more important than soil age alone in predicting the distribution of ecosystem properties across biomes. Thus, our work contextualizes the importance of soil age relative to other state factors that operate at large spatial scales, such as geology, climate, vegetation, and topography, which is an important step towards understanding the influence of long-term temporal dynamics on ecosystem properties across biomes.

Given that parent material, climate and vegetation type were the most important factors associated with variation in multiple ecosystem properties globally, we further investigated the direction of these effects and the potential mechanisms underlying the observed patterns (Figs. 3 and 4). Our results show that environmental context, rather than soil age, determine the values and the long-term temporal trajectories of ecosystem structure and function across biomes, and suggest that sandy, drier and non-forested ecosystems have lower levels of terrestrial ecosystem development than more mesic, forested ecosystem develop over other substrates (e.g., volcanic soils). As expected, parent material had a key influence on multiple ecosystem properties^1–3,15^. Sand-derived soils (e.g., MI, WA, QL) showed, on average, lower levels of total and available soil P, C stocks, soil respiration rates, microbial biomass and chemical index of alteration than soils developed on other substrates (e.g., volcanic or sedimentary) in globally distributed locations. Soils developed over sandy substrates supported faster increases in the C:P and N:P ratios during ecosystem development, and an overall flat or negative long-term temporal development in soil respiration, microbial biomass, C stocks, and available P, compared with other substrates. Our findings further showed that climate and vegetation play pivotal roles in determining the levels of ecosystem structure and function across biomes. Interestingly, irrespective of soil age, drylands, and non-forested ecosystems consistently had lower soil C stocks, plant productivity, percentage of tree cover, relative abundance of ectomycorrhizal fungi, and microbial biomass than more mesic and forested ecosystems (especially angiosperm forests). Moreover, mesic ecosystems had more acidic soils, greater levels of weathering (i.e., CIA), larger microbial biomass and higher tree cover, and higher C:P and N:P ratios than did drier environments, and supported steeper increases in plant productivity over time. Together, these results suggest that changes in the environmental context wherein ecosystems develop have important consequences for ecosystem structure and function, and could compromise the long-term development of terrestrial ecosystems in a drier and less-forested world.

Our findings provide further empirical evidence for the widely acknowledged paradigm in soil science and ecology that ecosystems of similar age can be at different points in their development^16–18^ for important ecosystem properties associated with biological activity such as soil N:P ratio, C stocks, and microbial biomass. In other words, the age of an ecosystem does not necessarily determine its level of development in structure and function. For example, on average, a 20,000-year old ecosystem from tropical forests from Hawaii showed 71 times more microbial biomass, and 13 times larger C stocks and higher N:P ratios, than arid soils of similar age from shrublands in Bolivia (BOV and BOS) and New Mexico (JOR; Fig. 9). Similarly, 4 million-year-old volcanic soils from Hawaii (HA) had 17 times more microbial biomass and 12 times higher N:P ratios than soils of similar age from Arizona (AZ; Fig. 9). This result is consistent with a recent regional-scale study of chronosequences across a climatic gradient in Western Australia^13^. Part of the reduced influence of time on ecosystem development in arid and colder environments might also be associated with processes of dust inputs and erosion^16^. Together, these findings indicate that environmental context regulates the structure and function of terrestrial ecosystems across biomes. Such findings are important because they provide insights into the potential responses of terrestrial ecosystems to ongoing global environmental change. Our results suggest that deforestation and transition from mesic to drier ecosystems may have important consequences for the long-term capacity of terrestrial lands to maintain critical goods and services, including C storage, soil fertility, and plant production.

Finally, despite the relatively low importance of soil age as an ecosystem driver across biomes, the data show that soil age can still explain an additional and unique portion of variation in the distribution of multiple ecosystem properties globally. Consequently, our results suggest that including information on soil age in modeling efforts can help improving global ecosystem models. Soil age was particularly important for predicting the distribution of key topsoil properties such as C:P and N:P ratios, pH, total P based on H_2_SO_4_ diggestions (as well as P digested using hydrofluoric acid, P-HF), microbial biomass and C stocks across biomes (Fig. 3). These variables also followed relatively consistent patterns during ecosystem development, with overall increases in N:P ratios, microbial biomass, and C stocks, and declines in total soil P and soil pH as ecosystems develop. These results are consistent with previous studies^6–13^, highlighting the importance of soil age in shaping patterns in some specific ecosystem properties within local soil chronosequences over time, and further suggest that some ecosystem properties follow fundamental patterns that can be generalized across terrestrial ecosystems worldwide. Even so, we stress that, for a subset of ecosystem properties, local environmental context still drove contrasting patterns for some important ecosystem properties. These ecosystem properties followed either biome- (e.g., soil C:N and fungal-to-bacterial ratios and chitin/sugar degradation) or site-dependent patterns (e.g., plant productivity and plant community composition), or lacked clear relationships with soil age (e.g., potential microbial activities for lignin and glucose respiration, and relative abundance of arbuscular mycorrhizal fungi). Many of these ecosystem properties are likely driven by idiosyncratic environmental and local conditions at a given location, rather than longer-term changes in ecosystems over time, although those longer-term changes can influence the state factors at each point in time.

Our work provides fundamental insights into the natural history of terrestrial ecosystems, and quantifies the relative importance of the state factors of ecosystem development in controlling multiple structural and functional properties across biomes. We found that soil age is a significant ecosystem driver, but only accounts for a relatively small proportion of variation in ecosystem structure and function across biomes. Soil age could help, for example, with the fine-tuning of global ecosystem models, and was an important ecosystem driver at the local scale where it supported some consistent patterns in the changes of ecosystem properties over time. However, our results also demonstrate that environmental context associated with parent material, climate and vegetation type, rather than soil age, play dominant roles in driving the values and long-term temporal trajectories structure and function of terrestrial ecosystems across biomes. For example, irrespective of soil age, drylands, and non-forested ecosystems consistently had lower measures of ecosystem function, and weaker increases in plant productivity over time, than did more mesic and forested ecosystems. Thus, variation in environmental contexts that result from global change factors, including shifts in precipitation, temperature, and vegetation, may substantially modify the conditions under which ecosystems develop, and slow down the longer-term development of terrestrial ecosystems in a drier, hotter and less-forested world^19,20^.

## Methods

### Cross-biome field survey and soil sample collection

Soil and vegetation data were collected using standardized protocols between 2016 and 2017 from 16 soil chronosequences (also known as substrate age gradients) located in nine countries and six continents (Fig. 1 and Supplementary Table 1). Soil chronosequences are often used to evaluate the changes in ecosystem structure and function over millennia because soil age for these locations is frequently known from geological surveys, models, and isotopic dating techniques (Fig. 1 and Supplementary Table 1). In these soil chronosequences, all other soil-forming factors except substrate age are kept relatively constant (i.e., current climate, vegetation, topography, and parent material), which permits the separation of the effects of time on ecosystem development from other ecosystem development state factors^1–3^.

Field surveys were conducted according to a standardized sampling protocol. We surveyed a 50 m × 50 m plot within each chronosequence stage, and within each quadrat, collected five composite surface soil samples from the surface 10 cm soil under the dominant vegetation types (e.g., trees, shrubs, grasses, etc.). Given the cross-biome nature of our study, we do not expect the timing (season) of sample collection to affect our results. Following field sampling, soils were sieved (<2 mm) and separated into two portions. One portion was air-dried and used for soil biochemical analyses. The other portion of the soil was immediately frozen at −20 °C for molecular analyses. This storage approach is commonly used in global surveys^21,22^. Our study includes 16 chronosequences, 87 plots, 261 transects, and 435 soil samples (Supplementary Table 3).

### State factors of ecosystem development

We used a total of 30 predictors to describe the five state factors of ecosystem development: soil age, climate, organisms, parent material, and topography (Supplementary Table 4). Parent material was meant to represent the geological material at each location, and was characterized using the information on chronosequence origin (e.g., volcanic, sand dunes, or sedimentary), lithology, and soil type (Supplementary Table 4). Climate was represented by annual mean values and seasonality of current temperature and precipitation (Supplementary Table 4). Vegetation was represented by whether a site was grassland, shrubland, coniferous or angiosperm forest (Supplementary Table 4). Topography was represented by elevation, slope, and aspect. Soil age was represented by semi-quantitative and quantitative categories (Supplementary Table 4). A complete list of these predictors, detailed information on their units, and a rationale on their significance for ecosystem structure and function are in Supplementary Table 4.

There is no single accepted way to describe soil age in ecological studies. Because of this, we characterized soil age using three complementary metrics: a quantitative index of soil age (years; log_10_-transformed), a semi-quantitative index of age (where samples were given three soil age categories: thousands of years, hundreds of thousand years and millions of years) and a qualitative soil age index (standardized soil age range from 0 to 1 calculated for each chronosequences) (see ref. ^13^ for a similar approach).

The vegetation type (coniferous and angiosperm forest, shrubland, grassland) and the geological substrate origin (glacier, sand dunes, sedimentary, or volcanic) were annotated at each location in situ. We included vegetation type (e.g., Angiosperm forest) as a predictor, and plant functional type composition (e.g., % of grasses; see below) as a response variable because all vegetation types include mixtures of plant functional types (Supplementary Table 1), and vegetation type explains only a minor proportion of the functional type composition of plant communities (Fig. 2).

We used information on substrate origin, lithology, and soil orders to describe the parent material in our soils (i.e., the geological material in which soil develops). We used the three most common substrate origins (volcanic, sand dunes, and sedimentary; Supplementary Table 4) in our global survey for downstream analyses. Lithology information was obtained from the PANGAEA database^23^. Soil US Department of Agriculture (USDA) class information was retrieved from the SoilGrids250m database^24^ at 250 m resolution. We used the top five most common lithology and USDA class soil types (Supplementary Table 3) in our global survey for downstream analyses.

We classified all chronosequences included in this study as cold (continental and polar weather), arid, temperate, and tropical using information of the Köppen climate classification^25^. Climatic variables were collected from the Worldclim database^26^. Climatic information included maximum and minimum temperature, temperature seasonality, mean diurnal temperature range, mean annual precipitation, precipitation seasonality, and climatic biome type (dryland and mesic ecosystems) (Supplementary Table 3).

Regarding topography, information on topographic elevation, slope, and aspect was retrieved from ref. ^27^ at 30 m resolution. We retrieved the averaged slope and aspect class for each location using this database (Supplementary Table 3).

### Current vs. past climates

Note that we used current climate^26^ information as our surrogate of climate. However, we also cross-validated this approach using paleoclimatic data (Worldclim database) from 64 soil chronosequences across the globe including the 16 chronosequences from this study (Fig. 1) and 48 other comparable soil chronosequences, using a global assessment of published data (Supplementary Fig. 1). Note that the current climate provided a good representation of the existing climate in multiple globally distributed soil chronosequences during hundreds of thousands of years, suggesting that locations where these chronosequences were developed experienced relatively small changes in precipitation and temperature patterns (Supplementary Fig. 5).

### Ecosystem functional and structural properties

We measured 32 functional and structural properties, which we collectively refer to as ecosystem properties. We included a wide range of above- and belowground pools and fluxes that represent both rapidly and slow-changing ecosystem properties. This allowed us to provide a comprehensive collection of variables representing the structure and function of terrestrial ecosystems worldwide. A description of the units, data availability, and data resolution (sample, plot, transect) for functional and structural properties is given in Supplementary Table 3. See Fig. 2 for a list of the considered structural and functional properties.

### Nutrient cycling

The concentrations of dissolved inorganic N and P were determined for all soil samples. The concentration of dissolved inorganic N was obtained from 0.5 M K_2_SO_4_ extracts using colorimetric analyses^21^. The concentration of Olsen P was determined from bicarbonate extracts using colorimetric analyses^21^. Soil Olsen P concentrations were positively correlated with other commonly used methods^28^ for estimating available P pool sizes (resin-P; *ρ* = 0.72, *P* < 0.001, *n* = 87).

### Water resources

We determined the water-holding capacity and potential infiltration of all soil samples. Soil water-holding capacity was determined in the lab using the funnel method as described in ref. ^29^. Potential water infiltration was determined in the lab using a similar method to that described in ref. ^30^.

### Mycorrhizal structure

We determined the relative abundance of soil ectomycorrhizal and arbuscular mycorrhizal fungi via amplicon sequencing using the Illumina MiSeq platform^14^. Soil DNA was extracted using the Powersoil® DNA Isolation Kit (MoBio Laboratories, Carlsbad, CA, USA) according to the manufacturer’s instructions^14^. A portion of the eukaryotic 18S (V9 region) rRNA genes^31^ was sequenced using the Euk1391f ((5′-GTACACACCGCCCGTC-3′)/EukBr (5′-TGATCCTTCTGCAGGTTCACCTAC-3′) primer set^14^. Bioinformatic processing was performed using a combination of QIIME^32^, USEARCH^33^, and UNOISE3^34^. Phylotypes (i.e., Operational taxonomic units; OTUs) were identified at the 100% identity level. Fungal guilds for fungal communities were identified using the FUNGUILD database^35^ focusing on probable and highly probable matches. The relative abundance (%) of ectomycorrhizal and arbuscular mycorrhizal fungi were calculated, from a 2000 reads/sample rarefied fungal OTU table^14^, as the proportion of 18S reads classified as mycorrhizal fungi (from unique matches) out of all fungal 18S reads. Taxa with mixed lifestyles were discarded.

### Carbon cycling

We determined the amount of C stocks and C fluxes in all soil samples as surrogates of climate regulation (soil-atmosphere CO_2_ feedbacks). The concentration of soil total organic C was determined by colorimetry after oxidation with a mixture of potassium dichromate and sulfuric acid^36^. Bulk density was determined in the field at the plot level as the average from three soil cores. Using this information, we calculated C stocks (kg C m^−2^) to 10 cm depth. Note that the concentrations of total organic C were strongly correlated with those of total N (*ρ* = 0.90; *P* < 0.001, *n* = 435) across samples, so we kept only soil C for statistical modeling. Soil respiration was determined on composite soil samples per plot by quantifying the CO_2_ released during 16 days from 1 g of soil sample incubated at 28 °C at 50% of water-holding capacity in 20-mL glass vials in the dark, following a 1-week pre-incubation^37^.

We measured three extracellular enzyme activities in all soil samples: activity of β-glucosidase (sugar degradation), *N*-acetylglucosaminidase (chitin degradation), phosphatase (phosphorus mineralization). Extracellular soil enzyme activities were measured using 1 g of soil by fluorometry^38^. We also determined substrate-induced respiration rates for lignin (lignin degradation) using the Microresp protocol^39^. In brief, samples with and without (basal respiration) lignin were incubated for 6 h and read at 570 nm. Lignin degradation was calculated as respiration in lignin less the basal respiration. Finally, the respiration of glucose was assayed through the incubation of soils in the above conditions for soil respiration (28 °C and 50% of water-holding capacity) and addition of 240 µg of ^13^C-glucose (99 atom% U-^13^C, Cambridge Isotope Laboratories, Tewksbury, Massachusetts, USA) dissolved in water to each vial^40^. CO_2_ production and its isotopic composition were then measured as described in ref. ^40^.

### Plant production and vegetation composition

We used the Normalized Difference Vegetation Index (NDVI) as our proxy for net plant primary productivity. This index provides a global measure of the “greenness” of vegetation across Earth’s landscapes for a given composite period, and thus acts as a proxy of photosynthetic activity and large-scale vegetation distribution. NDVI data were obtained from the Moderate Resolution Imaging Spectroradiometer (MODIS) aboard NASA’s Terra satellites (http://neo.sci.gsfc.nasa.gov/). We obtained plant productivity information (averaged monthly values between 2008 and 2017) at a resolution of 250 m. Within each 50 × 50 m plot, three 50-m parallel transects were established, spaced 25 m apart. The relative abundance of woody vs. non-woody plants was calculated using vegetation transect information, and used as a surrogate of the availability of wood resources (Supplementary Table 3). The percentage of cover by trees, shrubs, open areas, forbs, and grasses was measured in each of the three transects located within each plot (see above) using the line-intercept method^21^. The dominant vegetation in each location is available in Supplementary Table 1.

### Microbial biomass

Microbial biomass of soil was estimated from phospholipid fatty acids (PLFAs) extracted from freeze-dried soil samples and measured using the method described in ref. ^41^, as modified by ref. ^42^, to achieve high throughput analysis. The extracted fatty acid methyl esters were analyzed on an Agilent Technologies 7890B gas chromatograph with an Agilent DB-5 ms column (Agilent Technologies, CA, USA). The biomarkers selected to indicate total bacterial biomass are the PLFAs i15:0, a15:0, 15:0, i16:0, 16:1ω7, 17:0, i17:0, a17:0, cy17:0, 18:1ω7 and cy19:0, and the biomarker to indicate total fungal biomass is the PLFA 18:2ω6. Using the selected PLFA biomarkers, the biomass and the ratio of fungal and bacterial communities^43,44^ were calculated for each soil sample. Total microbial biomass includes the sum of all bacterial and fungal biomarkers plus that of other soil microbial biomarkers such as the eukaryotic C18:1w9. The fungal-to-bacterial ratio was calculated as total fungal PLFAs/total bacterial PLFAs, while the total microbial biomass for each soil sample was expressed as log_10_ (sum of all analyzed PLFAs).

### Soil properties

Soil pH was measured with a pH meter, in a 1:2.5 mass:volume soil and water suspension. Texture (% of fine fractions: clay + silt) was determined on a composite sample for each chronosequence stage^45^. Total element concentrations (P, Al, Ca, Na, K, Mg) were determined by inductively coupled plasma atomic emission spectroscopy (IPC-AES) after microwave digestion with aqua regia and hydrofluoric acid^46^. We refer to soil total P determined using this method as soil P-HF. In the case of soil P (in H_2_SO_4_), P was obtained using a SKALAR San++ Analyzer (Skalar, Breda, The Netherlands) after digestion with sulfuric acid (3 h at 415 °C)^21^. We refer to this soil total P as Soil total P- H_2_SO_4_. The chemical index of alteration (CIA) [Al/(Al + Ca+ Na + K)] and total base cation reserves (TBR) [Ca+ Na + K+ Mg])^47^ provide information on level of weathering. The CIA index is also referred to as aluminum saturation. Note that we used two types of weathering indexes (CIA and TBR) and soil total P forms because these measurements could be more meaningful for certain types of soils (e.g., CIA in volcanic and TBR in sedimentary soils) in providing complementary information on similar concepts.

### Soil stoichiometry

Soil total N concentrations were obtained using a SKALAR San++ Analyzer (Skalar, Breda, The Netherlands) after digestion with sulfuric acid (3 h at 415 °C)^21^. Soil C:N, N:P and C:P ratios were calculated using the information on total organic C and soil P- H_2_SO_4_ for consistency with ref. ^14^ which were analyzed as explained above.

### Meta-analysis of published data

In addition to the surveyed 16 soil chronosequences, we conducted a meta-analysis (i.e., a statistical synthesis of results combining data from multiple separate studies) including information from several other independent, but comparable (centuries to millennia), soil chronosequences and important ecosystem properties (Supplementary Fig. 1; Table 2 and Supplementary Methods 1 and 2) to further validate some of our conclusions. We used the SCOPUS database (accessed August 2016) to extract data from published reports, articles, and reviews on the effects of ecosystem development on soil properties and vegetation. The following keyword combinations were used: “chronosequence” AND (“carbon” OR “nitrogen” OR “phosphorus” OR “biomass” OR “diversity”). Within these references, studies were included in our analyses only if they represented soil chronosequences that spanned longer time periods (i.e. centuries to millennia or longer). We obtained enough information for soil C stocks (calculated using bulk density information when available), total P, pH, texture (clay + silt) and soil C:N ratios. These variables also had the advantage that they are often measured with very similar methods and contain comparable information in terms of units etc. We used comparable surface soil data from the mineral surface horizons for our analyses (top ~10 cm). We excluded the 16 soil chronosequences surveyed in this study, to ensure independence of both databases. A total of 48 soil chronosequences were retained from our literature search (see Supplementary Fig. 1; Table 2 and Supplementary Methods 1 for a complete list of chronosequences studies).

### Variation partitioning modeling

We then used Variation Partitioning Modeling^48^ to quantify the relative importance of soil age, climate, vegetation type, parent material type, and topography (Supplementary Table 4) in regulating 32 ecosystem properties. Specifically, this analysis allowed us to identify the unique and shared portion of the variation in the distribution of multiple ecosystem functional and structural properties explained by the five state factors that determine ecosystem properties. This approach is recommended specifically to deal with among-group multicollinearity, as it partitions the variance in a given response variable (ecosystem property) that is attributed to a particular group of predictors (a group of variables representing a given state factor; e.g., climate) from that variance shared among all predictors (all-state factors)^48^. Note that adjusted coefficients of determination in multiple regression/canonical analysis can, on occasion, take negative values^48^. Negative values in the variance explained for a group of predictors on a given response variable are interpreted as zeros, and correspond to cases in which the explanatory variables explain less variation than random normal variables would^48^.

We used the varpart function from the “vegan” R^49^ package to run these analyses. The original package is designed to evaluate the unique portions of variations in a given variable (e.g., function 1) explained by four explanatory matrices (groups of statistical predictors). We bypassed this issue by running each model two times. First, we ran our models with two explanatory tables (soil age and the remaining state factors). Using this run, we determined the amount of variation explained by soil age alone and shared with the environment. We then further partition the fraction of variation uniquely explained by the combination of state factors into unique and shared fractions explained by climate, vegetation type, parent material, and topography.

### Partial correlations

We complemented our Variation Partitioning modeling by conducting further partial (Spearman) correlations to evaluate the associations between 30 single variables within the climate, vegetation type, parent material and topography (Supplementary Table 4) with multiple ecosystem properties (Supplementary Table 4). These analyses were statistically controlled by three complementary soil age metrics (Supplementary Table 4) described above. We used the pcor function from the “ppcor” R^50^ package to run these analyses.

### Changes in ecosystem structure and function within soil chronosequences

We further employed non-parametric Spearman rank correlations to further explore the potential associations between soil age (i.e., chronosequences stage) and 32 ecosystem properties. By using Spearman correlations, we aimed to identify the most important trends in our results. Spearman rank correlations measure the strength and direction of the association between two ranked variables. Spearman rank correlations do not require the normality of data or homogeneity of variances. Moreover, linearity is not strictly an assumption of these correlations (they can be run on a non-monotonic relationship to determine whether there is a monotonic component to the association, and therefore used to identify the most important trends between two variables). In addition, unlike Pearson correlations, Spearman rank correlations can be used to associate two variables regardless of whether they are ordinal, interval, or ratio. Spearman correlations have been used in many studies to identify major associations between soil age and ecosystem properties^4,14,51^. Again, for consistency with the previous work^4,14,51^, we used chronosequence stage as our surrogate for soil age in these analyses.

### Reporting summary

Further information on research design is available in the Nature Research Reporting Summary linked to this article.

## Supplementary information

Supplementary Information

Reporting Summary

## Data Availability

Ecosystem structural and functional data from the global field survey are publicly available in Figshare^52^.

## Code availability

Many of the numerical analyses included in this article do not have an associated code. Used codes are available in refs. ^49,50^.
