# Supplementary material for: The influence of soil age on ecosystem structure and function across biomes
Source: Nat Commun. 2020 Sep 18;11:4721. doi: 10.1038/s41467-020-18451-3 (PMC7501311; doi:10.1038/s41467-020-18451-3)
Supplement: Supplementary file 1 — Supplementary Information [file 41467_2020_18451_MOESM1_ESM.pdf]

nature COMMUNICATIONS

Supplementary information for

The influence of soil age on ecosystem structure and function across biomes

Author for correspondence:

Manuel Delgado-Baquerizo. E-mail: M.DelgadoBaquerizo@gmail.com

This PDF file includes:

Supplementary Figures 1-6
Supplementary Tables 1-7
Supplementary Methods 1 and 2

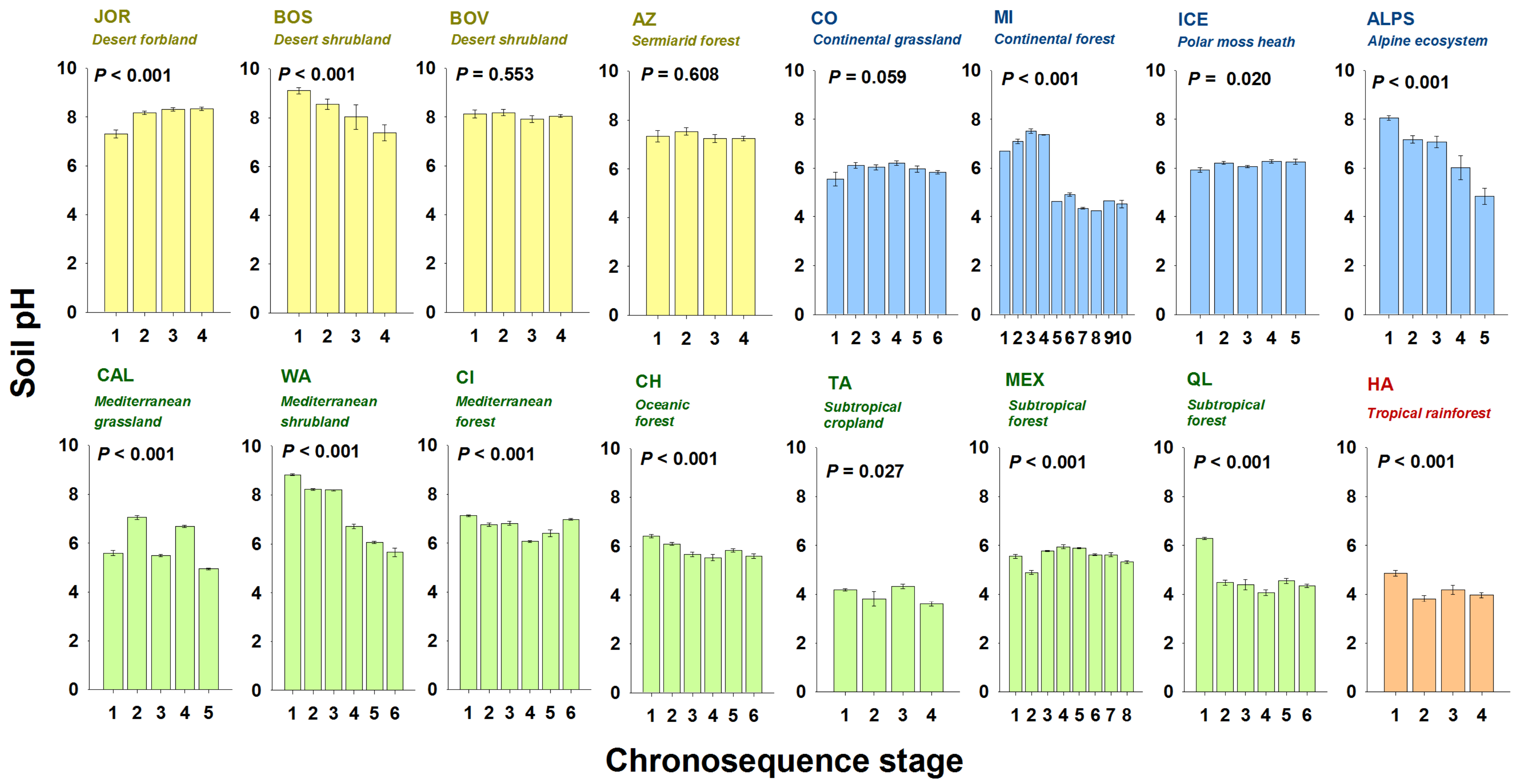

Supplementary Figure 1 | Changes soil pH during ecosystem development (mean ± SE). PERMANOVA P values are shown. Chronosequence stage was included as a fixed factor in these analyses (n = 5).

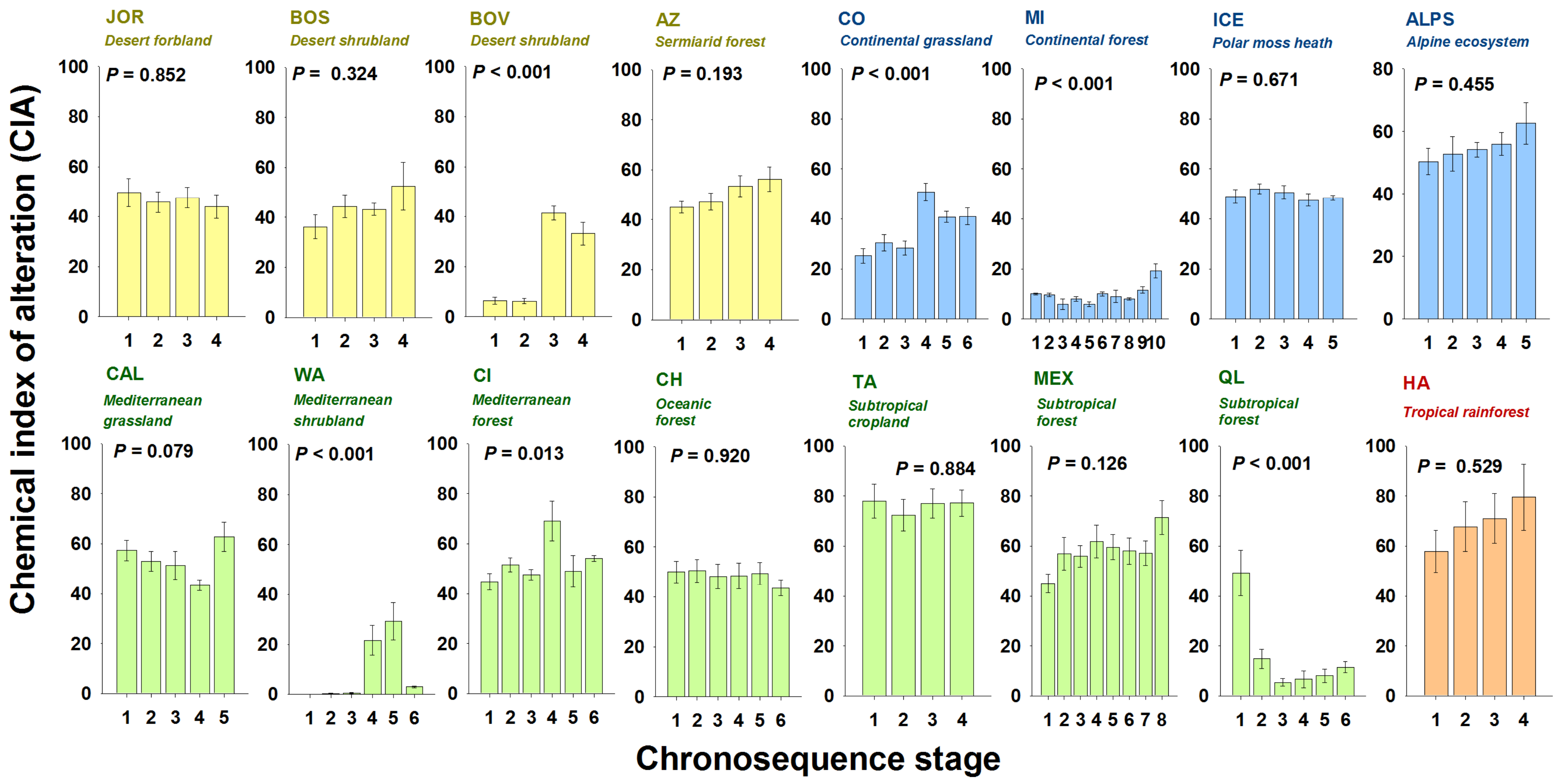

Supplementary Figure 2 | Changes in the chemical index of alternation during ecosystem development (mean ± SE). PERMANOVA P values are shown. Chronosequence stage was included as a fixed factor in these analyses (n = 5). CIA = %.

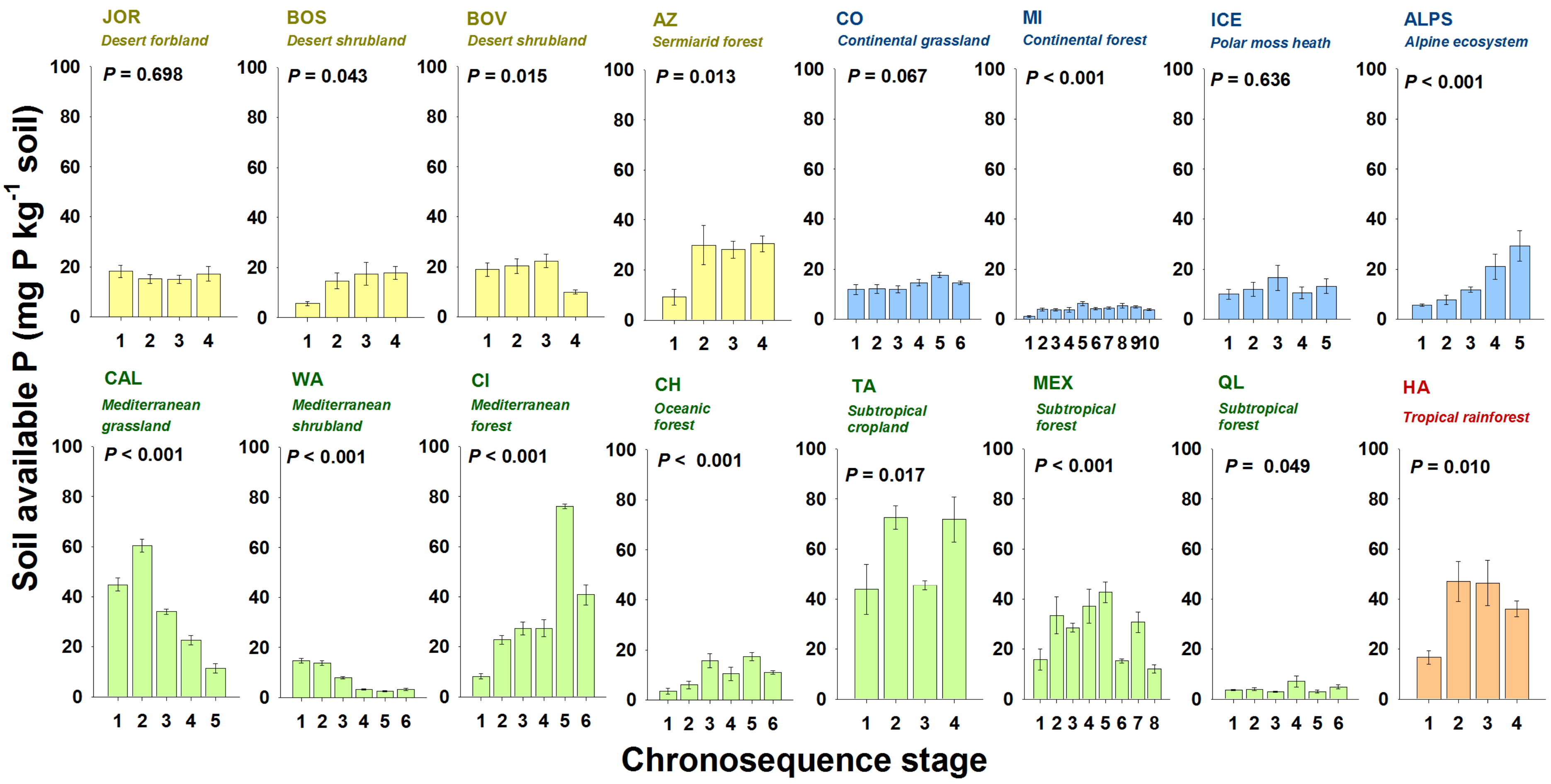

Supplementary Figure 3 | Changes in soil available P during ecosystem development (mean ± SE). PERMANOVA P values are shown. Chronosequence stage was included as a fixed factor in these analyses (n = 5).

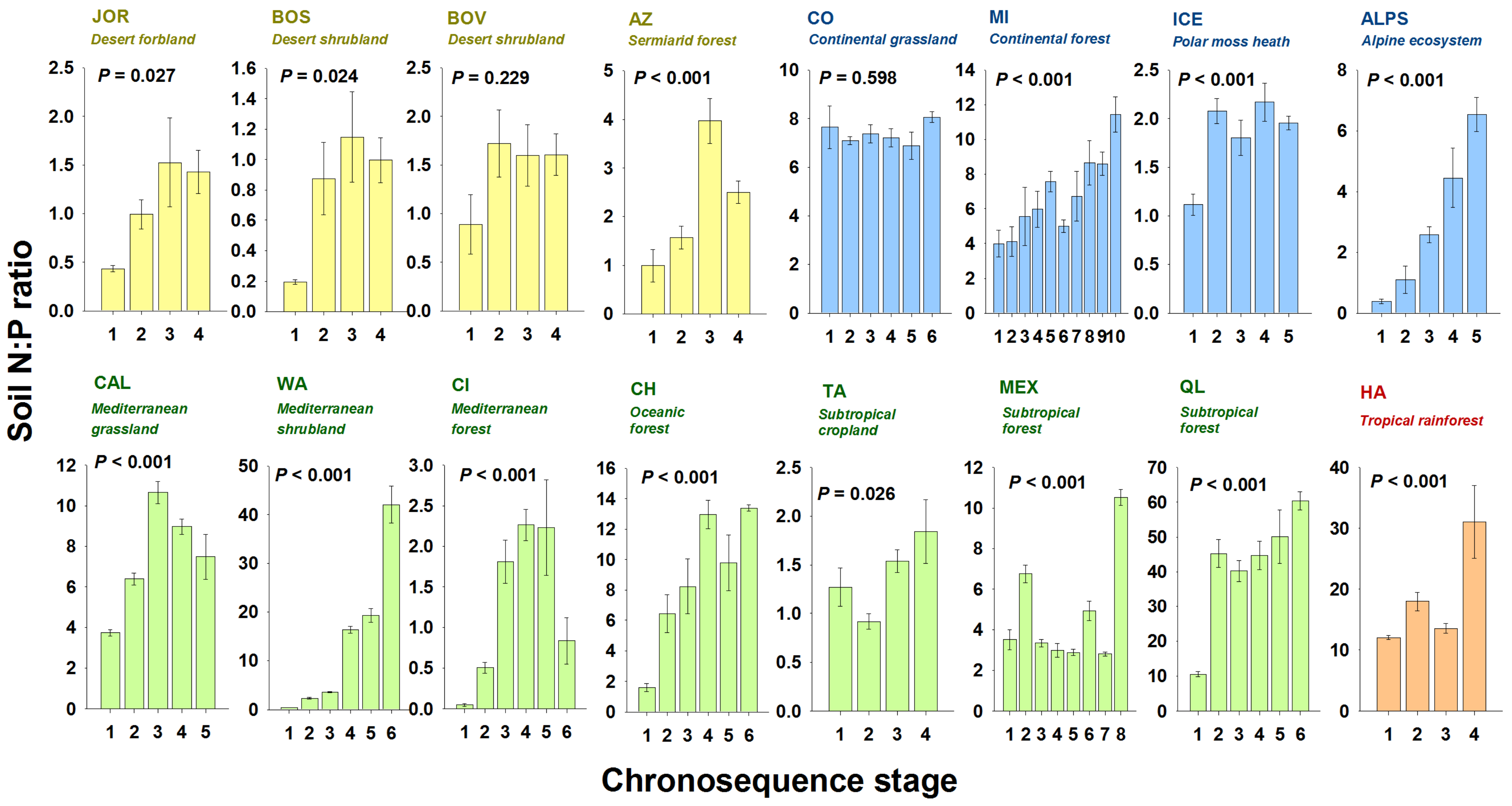

Supplementary Figure 4 | Changes in the soil N:P ratio during ecosystem development (mean ± SE). PERMANOVA P values are shown. Chronosequence stage was included as a fixed factor in these analyses (n = 5).

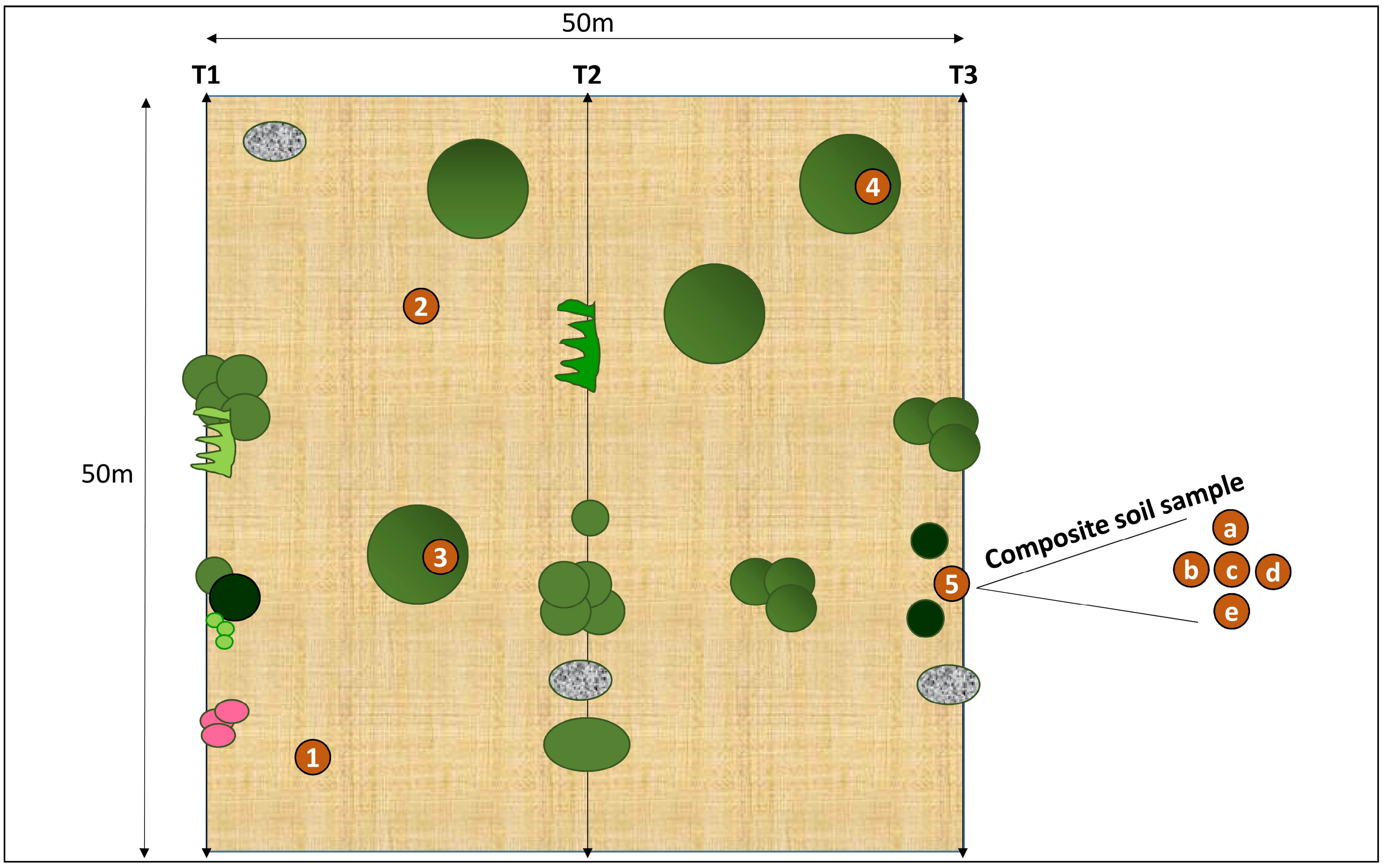

Supplementary Figure 5 | Graphical representation of our sampling design implemented at each stage for each chronosequence. T = transect. Different circles represent different types of vegetation.

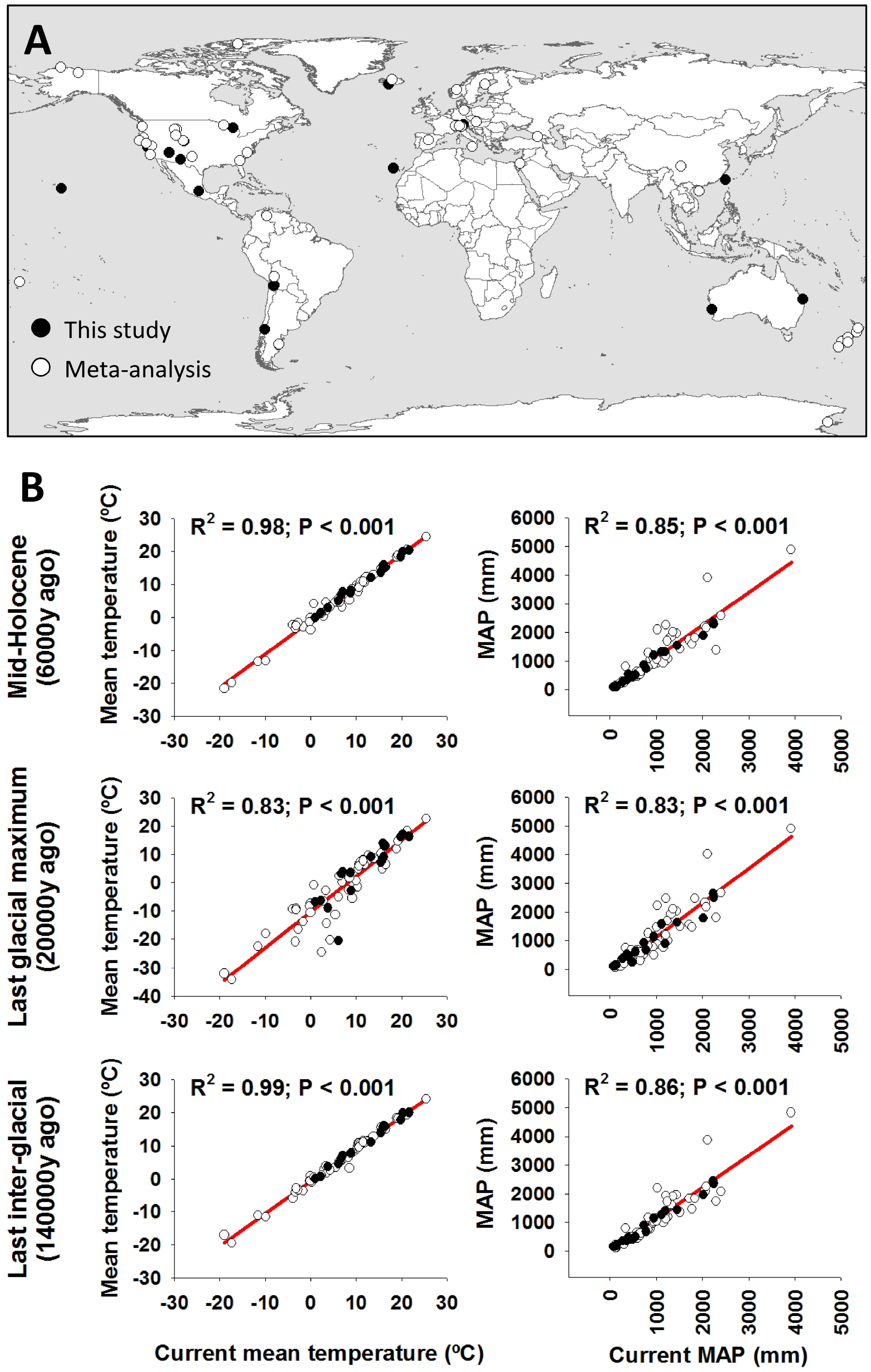

Supplementary Figure 6 | Relationship between current and past climates across 64 chronosequences. Panel A shows the location of the 64 soil chronosequences included in this study. Panel B includes the regression between current and past precipitation (MAP) and temperature for the 64 chronosequences (16 soil chronosequences in this study, and 48 soil chronosequences a meta-analysis) across three periods of time (6k, 20k and 140k years ago). Black circle = This study; White circle = Meta-analysis. P values were derived from ordinary least square regressions.

Supplementary Table 1 | Dominant vegetation community composition in each of the stages for the 16 soil chronosequences included in this study. See Fig. 1 for the location of these chronosequences. A previous reference including information on these chronosequences can be found at the bottom of this supplementary table.

Name	Stage	Dominant vegetation	Age (years)
ALPS[a]	1	*Saxifraga azoides, Saxifraga oppositifolia, Poa alpina, Linaria alpina, Artemisia gentipi*	10
	2	*Trifolium pallescens, Campanula scheuchzeri, Saxifraga oppositifolia, Saxifraga aizoides*	45
	3	*Kobresia myosuroides, Agrostis alpina, Alchemilla fissa, Trifolium pratense* spp., *Nivale*	125
	4	*Avenula versicolor, Carex sempervirens, Festuca halleri, Anthoxanthum alpinum*	10000
	5	*Fagus sylvatica, Abies alba, Acer pseudoplatanus, Picea abies, Quercus robur*	120000
AZ[b]	1	*Juniperus monosperma, Pinus edulis, Bouteloua gracilis*	900
	2	*Juniperus monosperma, Pinus edulis, Bouteloua gracilis*	55000
	3	*Juniperus monosperma, Pinus edulis, Bouteloua gracilis*	750000
	4	*Juniperus monosperma, Pinus edulis, Bouteloua gracilis*	3000000
BOS[c]	1	*Astragalus pusillus, Atriplex imbricata, Baccharis boliviensis, Baccharis tola, Ephedra breana, Haplopappus rigidus, Junellia seriphioides, Lycium chanar, Opuntia boliviensis*	25
	2	*Fabiana densa, Atriplex imbricata, Baccharis boliviensis, Lycium chanar, Baccharis tola, Haplopappus rigidus, Hoffmannseggia minor, Junellia seriphioides, Mutisia ledifolia, Nassella curviseta*	11400
	3	*Atriplex imbricata, Baccharis boliviensis, Haplopappus rigidus, Junellia seriphioides, Lycium chanar, Mutisia ledifolia, Nassella curviseta, Trichocereus atacamensis*	14100
	4	*Atriplex imbricata, Baccharis boliviensis, Cheilanthes ternifolia, Diplostephium cinereum, Ephedra breana, Fabiana densa, Lycium chanar, Mutisia ledifolia, Senecio dryophyllus, Senecio nutans, Stevia sp., Trichocereus atacamensis*	20000
BOV[c]	1	*Adesmia spinosa, Atriplex imbricata, Chuquiraga atacamensis. Frankenia triandra, Sisymbrium sp., Nassella curviseta*	25
	2	*Opuntia boliviensis, Acantholippia punensis, Atriplex imbricata, Chuquiraga atacamensis, Ephedra breana, Senecio dryophyllus, Sisymbrium sp.*	11400
	3	*Acantholippia punensis, Adesmia spinosa, Atriplex imbricata, Chuquiraga atacamensis, Nassella curviseta*	14100
	4	*Acantholippia punensis, Atriplex imbricata, Chuquiraga atacamensis, Senecio dryophyllus*	20000
CAL[d]	1	*Populus fremontii, Helianthus annuus, Amaranthus albus*	100
	2	*Quercus lobata, Silybum marianum, Hordeum murinum L*	3000
	3	*Festuca californica*	30000
	4	*Rytidosperma penicillatum*	600000
	5	*Festuca bromoides, F. myuros, Bromus hordaceous, Bromus diandrus*	3000000
CH[e]	1	*Gaultheria pumila, Racomitrium lanuginosum*	60
	2	*Lomatia hirsuta, Austrocedrus chilensis*	266
	3	*Araucaria araucana, Nothofagus antarctica*	776
	4	*Nothofagus dombeyi, Araucaria araucana*	3470
	5	*Nothofagus dombeyi, N. obliqua, N. alpina*	60000
	6	*Nothofagus dombeyi, N. alpina*	5000000
CI[f]	1	*Pinus canariensis*	525
	2	*Pinus canariensis, Erica arborea, Pterocephalus porphyranthus*	6000
	3	*Pinus canariensis, Adenocarpus viscosus, Chamaecytisus proliferus, Erica arborea*	40000
	4	*Pinus canariensis, Adenocarpus viscosus*	600000
	5	*Pinus canariensis, Cistus symphytifolius*	1100000
	6	*Pinus canariensis, Cistus symphytifolius*	1700000
CO[f]	1	*Juncus arcticus, Andropogon gerardii, Panicum virgatum*	5000
	2	*Andropogon gerardii, Panicum virgatum*	140000
	3	*Panicum virgatum, Poa compresa, Andropogon gerardii*	240000
	4	*Chrysopsis sp., Andropogon gerardii, L. cinquefoil*	640000
	5	*Andropogon gerardii, M. Burgia, Poa compresa,*	1000000
	6	*Andropogon gerardii, Poa compresa, M. Burgia*	2000000
HA[g]	1	*Metrosideros polymorpha, Morella faya, Vaccinium calycinum, Ilex anomala, Cheirodendron trigynum, Cibotium glaucom, Hedychium gardnerianum, Isoetes sp. (grass), Coprosma sp., Myrsine lessertiana, Dicranopteris linearis, Machaerina angustifolia, Anemone hupehensis,*	300
	2	*Metrosideros polymorpha, Cheirodendron trigynum, Cibotium glaucom, Cibotium menziesii, Ilex anomala, Freycinetia arborea, Astelia menziesii, Melicope clusiifolia, Vaccinium calycinum, Nephrolepis sp., Asplenium* spp. *(multi), Athyrium microphyllum, Ilex myrtifolia, Peperomia sp., Polypodium sp.,*	20000
	3	*Metrosideros polymorpha, Cibotium glaucom, Cibotium menziesii, Hedychium gardnerianum, Vaccinium calycinum, Cheirodendron trigynum, Psidium cattleianum, Dicranopteris linearis, Asplenium sp., Melicope clusiifolia, Myrsine sandwicensis, Elaphoglossum sp., Polygonum punctatum Tibouchina herbacea, Peperomia sp., Psilotum nudum*	150000
	4	*Metrosideros polymorpha, Hedychium gardnerianum, Dicranopteris linearis, Pittosporum gayanum, Psidium cattleianum, Astelia menziesiana, Morella faya, Vaccinium meyenianum, Smilax hawaiensis, Elaphoglossum spp, Elaeocarpus bifidus, Clerodendrum sp., Alyxia oliviformis,*	4100000
ICE[h]	1	*Racomitrium lanuginosum; Empetrum nigrum; Stereocaulon vesuvianum*	172
	2	*Racomitrium lanuginosum; Empetrum nigrum; Arctostaphylos uva-ursi*	463
	3	*Racomitrium lanuginosum; Betula nana; Hylocomium splendens; Empetrum nigrum; Arctostaphylos uva-ursi*	628
	4	*Racomitrium lanuginosum; Salix phylicifolia; Empetrum nigrum; Hylocomium splendens*	717
	5	*Racomitrium lanuginosum; Empetrum nigrum; Betula nana; Hylocomium splendens*	859
JOR[i]	1	*Opuntia phaeacantha. var., Boerhavia* spp., *Eragrostis lehmanniana.*	1100
	2	*Sporobolus contractus, Muhlenbergia porteri, Larrea tridentata, Ephedra trifurca.*	2200
	3	*Boerhavia* spp., *Larrea tridentata .*	8000
	4	*Boerhavia* spp., *Ephedra trifurca Torr., Erioneuron pulchellum*	25000
MEX[j]	1	*Pinus montezumae, Bacharis conferta, Alnus firmifolia, Penstemon sp.*	1000
	2	*Abies religiosa, Arbutus xalapensis, Pinus herrerae, Bacharis conferta, Pinus montezumae, Penstemon sp., Bacharis conferta*	1835
	3	*Pinus montezumae, Pinus pseudostrobus, Alnus firmifolia, Quercus laurina,*	3800
	4	*Pinus montezumae, Alnus firmifolia*	6200
	5	*Pinus montezumae, Bacharis conferta, Buddleja parviflora*	8000
	6	*Pinus patula, Alnus firmifolia, Pinus montezumae, Senecio sp.*	10000
	7	*Pinus ayacahuite, Pinus pseudostrobus, Pinus montezumae*	30500
	8	*Pinus montezumae, Abies religiosa, Quercus laurina, Penstemos sp., Bacharis conferta*	100000
MI[k]	1	*Amophilous breviligulata, Agropyron dasystachium, Cerisium pitheri, Arctostaphylos uva-ursi*	73
	2	*Amophilous breviligulata, Agropyron dasystachium, Cerisium pitheri, Arctostaphylos uva-ursi, Schizachyrium scoparium*	113
	3	*Arctostaphylos uva-ursi, Juniperus communis, Pinus strobus*	163

	4	*Pteridium aquilinum, Pinus resinosa, Abies sp.*	243
	5	*Gaultheria procumbens, Pinus resinosa,*	485
	6	*Abies balsamea, Pinus resinosa, Juniperus communis*	863
	7	*Abies balsamea, Pinus resinosa, Pinus strobus*	1400
	8	*Pinus resinosa, Vaccinium myrtilloides, Gaultheria procumbens*	2500
	9	*Pinus resinosa, Vaccinium myrtilloides, Gaultheria procumbens*	3200
	10	*Pinus resinosa, Pinus strobus, Gaultheria procumbens*	4000
QL[l]	1	*Eucalyptus tessellaris, Angophora costata, Eucalyptus intermedia, Casuarina littoralis, Melaleuca quinquenerva, Banksia integrifolia, Banksia serrata, Macrozamia* spp., *Acacia aulacocarpa, Acacia flavescens, Cassytha paniculata, Gahnia sieberiana,*	3600
	2	*Eucalyptus tessellaris, Angophora costata, Eucalyptus intermedia, Casuarina littoralis, Melaleuca quinquenerva, Banksia integrifolia, Banksia serrata, Macrozamia* spp., *Acacia aulacocarpa, Acacia flavescens, Cassytha paniculata, Gahnia sieberiana,*	6700
	3	*Eucalyptus tessellaris, Angophora costata, Eucalyptus intermedia, Casuarina littoralis, Melaleuca quinquenerva, Banksia integrifolia, Banksia serrata, Macrozamia* spp., *Acacia aulacocarpa, Acacia flavescens, , Cassytha paniculata, Gahnia sieberiana,*	134000
	4	*Eucalyptus tessellaris, Angophora costata, Eucalyptus intermedia, Casuarina littoralis, Melaleuca quinquenerva, Banksia integrifolia, Banksia serrata, Macrozamia* spp., *Acacia aulacocarpa, Acacia flavescens, Cassytha paniculata, Gahnia sieberiana,*	176000
	5	*Eucalyptus tessellaris, Angophora costata, Eucalyptus intermedia, Casuarina littoralis, Melaleuca quinquenerva, Banksia integrifolia, Banksia serrata, Macrozamia* spp., *Acacia aulacocarpa, Acacia flavescens, Cassytha paniculata, Gahnia sieberiana,*	324000
	6	*Eucalyptus tessellaris, Angophora costata, Eucalyptus intermedia, Casuarina littoralis, Melaleuca quinquenerva, Banksia integrifolia, Banksia serrata, Macrozamia* spp., *Acacia aulacocarpa, Acacia flavescens, Cassytha paniculata, Gahnia sieberiana,*	716000
TA[m]	1	*Tea camellia*	28000
	2	*Tea camellia*	105000
	3	*Tea camellia*	322000
	4	*Tea camellia*	399000
WA[n]	1	*Acacia cyclops, Acacia rostellifera, Scaevola crassifolia, Olearia axillaris, Spyridium globulosum*	100
	2	*Melaleuca systena, Acacia lasiocarpa, Acacia rostellifera*	1000
	3	*Melaleuca systena, Acacia lasiocarpa, Acacia rostellifera*	6500
	4	*Melaleuca systena, Banksia leptophylla, Calothamnus quadrifidus*	120000
	5	*Banksia menziesii, Banksia attenuata, Mesomelaena pseudostygia, Hibbertia hypericoides*	480000
	6	*Banksia menziesii, Jacksonia floribunda, Banksia leptophylla*	2000000

[a]Kaufmann, R. (2001). Invertebrate Succession on an Alpine Glacier Foreland. Ecology 82, 2261-2278.

[b]Selmants, P.C., Hart S.C. (2008). Substrate age and tree islands influence carbon and nitrogen dynamics across a retrogressive semiarid chronosequence. Global Biogeochemical Cycles 22, GB1021.

[c]Alfaro, F.D. et al. (2017). Microbial communities in soil chronosequences with distinct parent material: The effect of soil pH and litter quality. Journal of Ecology 105, 1709-1722.

[d]Doetterl S. et al. (2019). Links among warming, carbon and microbial dynamics mediated by soil mineral weathering. Nature Geoscience 11, 589-593.

[e]Pérez, C.A. et al. (2017). Biological nitrogen fixation in a post-volcanic chronosequence from south-central Chile. Biogeochemistry 132, 23-36.

[f]Delgado-Baquerizo M. et al. (2019). Changes in belowground biodiversity during ecosystem development. PNAS 116, 6891-6896.

[g]Chadwick, O.A. et al. (1999). Changing sources of nutrients during four million years of ecosystem development. Nature 397, 491–497.

[h]Cutler, N.A. et al. (2008). The spatiotemporal dynamics of a primary succession. Journal of Ecology 96, 231-246.

[i]Lajtha K., Schlesinger W.H. (1988). The Biogeochemistry of Phosphorus Cycling and Phosphorus Availability Along a Desert Soil Chronosequence. Ecology 69, 24-39.

[j]Peña-Ramírez, V.M. et al. (2015). Rates of pedogenic processes in a chronosequence of volcanic ash soils of Central Mexico. Quaternary International 376, 19-33.

[k]Williams, M.A. et al. (2013). Bacterial communities in soil mimic patterns of vegetative succession and ecosystem climax but are resilient to change between seasons. Soil Biology and Biochemistry 57, 749-757.

[l]Walker, J. et al. (2018). Dating the Cooloola coastal dunes of South-Eastern Queensland, Australia. Marine Geology 398, 73–85.

[m]Tsai, H. et al. (2006). A river terrace soil chronosequence of the pakua tableland in central Taiwan. Soil Science 171, 167-179.

[n]Laliberté, E. et al. (2014). Environmental filtering explains variation in plant diversity along resource gradients. Science 345, 1602-1605.

Supplementary Table 2 | Main characteristics for the 48 soil age chronosequences included in the result cross-validations for study. See Appendix S1 for a list of paper including this meta-data. See Fig. 3A for the location of these chronosequences.

Chronosequence	Climate	Age range (years)	Substrate origin	Region	Vegetation type
Altamaha and Ohoopee River Valley, USA	Temperate	0-77000	Dune	Middle Latitudes	Grassland
Arklio Glacier, Canada	Cold	300-17000	Glacier	Polar circle	Unvegetated
Barrow Peninsula Canada	Cold	25-3750	Sedimentary	Polar circle	Grassland
Cowlitz River, WA, USA	Temperate	250-1200000	Sedimentary	Middle Latitudes	Forest
Damma Glacier, Switzerland	Cold	7-3000	Glacier	Middle Latitudes	Shrubland
Danube floodplain, Austria	Temperate	2-7500	Sedimentary	Middle Latitudes	Grassland
Elbe River, Germany	Temperate	100-6000	Sedimentary	Middle Latitudes	Forest
Franz Josef Glacier, New Zealand	Temperate	1-120000	Glacier	Middle Latitudes	Forest
Gletsch, Switzerland	Cold	150-10500	Glacier	Middle Latitudes	Grassland
Colorado Front Range, USA	Cold	225-11000	Glacier	Middle Latitudes	Forest
Golfo San Jorge, Argentina	Arid	1376-6238	Volcanic	Middle Latitudes	Shrubland
Haast dune system, New Zealand	Temperate	181-6500	Dune	Middle Latitudes	Shrubland
Hailuogou chronosequence, China	Temperate	0-1400	Glacier	Middle Latitudes	Forest
Hainan Island, China	Tropical	90-2300000	Volcanic	Tropics	Forest
Henares River, Spain	Temperate	120000-750000	Sedimentary	Middle Latitudes	Grassland
Kane Alluvial Fans, USA	Arid	5000-585000	Sedimentary	Middle Latitudes	Grassland
Lubbock Lake, USA	Arid	175-4750	Sedimentary	Middle Latitudes	Grassland
Makhtesh Ramon crater, Israel	Arid	60000-443000	Volcanic	Middle Latitudes	Forest
Manawatu, New Zealand	Temperate	0-10000	Dune	Middle Latitudes	Grassland
Supsa River basin, USA	Cold	1000-300000	Sedimentary	Middle Latitudes	Forest
Mono Lake	Temperate	47-2400	Dune	Middle Latitudes	Shrubland
Montana, USA	Cold	7_2000	Sedimentary	Middle Latitudes	Grassland
Morteratsch, Switzerland	Cold	1-12500	Glacier	Middle Latitudes	Grassland
Mt Shasta, USA	Temperate	27-1200	Volcanic	Middle Latitudes	Forest
Mururata Mountain, Bolivia	Temperate	900-20000	Glacier	Tropics	Grassland
Oberaar, Switzerland	Cold	150-11450	Glacier	Middle Latitudes	Grassland
Reefton, New Zealand	Temperate	1000-130000	Volcanic	Middle Latitudes	Forest
Rhone, Switzerland	Cold	150-11600	Glacier	Middle Latitudes	Grassland

San Francisco California, USA	Temperate	100000-500000	Sedimentary	Middle Latitudes	Forest
Schmadri, Switzerland	Cold	3500-11500	Glacier	Middle Latitudes	Grassland
Sicily, Italy	Temperate	60-31700	Volcanic	Middle Latitudes	Forest
Sierra Nevada Nat. Park, Venezuela	Temperate	1-11540	Volcanic	Tropics	Forest
South Eureka, USA	Temperate	3900-240000	Sedimentary	Middle Latitudes	Grassland
Storbreen glacier, Norway	Cold	70-9000	Glacier	Middle Latitudes	Grassland
The Lambardalur Cirque, Iceland	Cold	250-9000	Glacier	Middle Latitudes	Grassland
The McMurdo Valleys, Antarctica	Cold	14-4155	Glacier	Polar circle	Unvegetated
Tonga Islands, Tonga	Tropical	100-100000	Volcanic	Tropics	Forest
Toolik Lake, USA	Cold	5-4800000	Glacier	Polar circle	Forest
Val di Rabbi, Switzerland	Cold	4730-17300	Glacier	Middle Latitudes	Forest
Val Mulix, Switzerland	Cold	6900-19200	Glacier	Middle Latitudes	Grassland
Västerbotten, Sweden	Cold	90-6800	Glacier	Middle Latitudes	Forest
Ventura, USA	Temperate	700-80000	Sedimentary	Middle Latitudes	Grassland
Volcanic field, NZ	Temperate	1750-14000	Volcanic	Middle Latitudes	Forest
Waitutu, New Zealand	Temperate	3000-291000	Sedimentary	Middle Latitudes	Shrubland
Whataroa and Poerua Rivers, NZ	Temperate	14000-60000	Glacier	Middle Latitudes	Forest
Wind River Range, USA	Arid	150-1200000	Sedimentary	Middle Latitudes	Grassland
Wisconsin Glacier, USA	Cold	9500-13500	Glacier	Middle Latitudes	Forest
Yuba Rivers, Sacramento, USA	Temperate	0-1600000	Sedimentary	Middle Latitudes	Grassland

Supplementary Table 3 | List of ecosystem properties. Replicate-level = 435 samples (5 replicates/plot). Transect level = 261 transects (3 transects/plot). Plot-level = 87 plots.

#	Group	Ecosystem properties	Details	n	Rationale
1	**Soil properties**	**Chemical index of alteration (CIA index)**	$Al_2O_3/(Al_2O_3 + CaO + Na_2O + K_2O)$ (molecular proportions in %, based on total elements and not corrected for carbonates and apatite)	Replicate-level	Soil properties drive ecosystem change and are key regulators of ecosystem functioning. Texture refers to the size of the particles that make up the soil (clay, silt and sand). Soil pH is a measure of the acidity and alkalinity in soils. Total soil P provides information on the bedrock-associated P stocks. The total base cation reserve and the chemical index of alteration (aluminum saturation) informs on the degree of soil weathering, because Ca, Mg, K and Na are largely washed out during soil chemical alteration relative to other elements such as Al.
2		**Total base cation reserves (TBR index)**	Ca+ Na + K + Mg ($cmol_c\ kg^{-1}$)	Replicate-level	
3		**Fine texture**	Clay+silt (%)	Plot-level	
4		**Soil pH**	Soil pH	Replicate-level	
5		**Soil total P-H_2SO_4**	Total soil P-H_2SO_4 extraction (mg P kg^{-1} soil)	Replicate-level	
6		**Soil total P-HF**	Total soil P- hydrofluoric acid (-PH) extraction (mg P kg^{-1} soil)	Replicate-level	
7	**Soil stoichiometry**	**Soil C:P ratio**	Soil C:P ratio	Replicate-level	Ecosystem stoichiometry, and notably soil C:N:P stoichiometry, informs on the relative balance of carbon, nitrogen and phosphorus in the soil system. Ecosystem stoichiometry is a major regulator of important ecosystem functions such as organic matter mineralization and nutrient uptake.
8		**Soil N:P ratio**	Soil N:P ratio	Replicate-level	
9		**Soil C:N ratio**	Soil C:N ratio	Replicate-level	
10	**Water resources**	**Potential infiltration**	Potential water infiltration (ml water s^{-1})	Replicate-level	Hydrological functions regulate the availability and quality of fresh water via water purification (e.g., infiltration) and retention (water holding capacity). Water holding capacity provides information on the potential capacity of a given soil to retain water and potential water infiltration provide a measurement of the infiltration capacity for a given soil. Hydrological processes regulate the availability of hydric resources for plants, animals and microbes.
11		**Water holding capacity**	Soil water holding capacity (%)	Replicate-level	
12	**Nutrient cycling**	**Available P**	Soil available phosphorus (mg P kg^{-1} soil)	Replicate-level	Nutrient recycling, represented by soil inorganic N and P pools, is one of the most important processes controlling the productivity of plants and microbes in terrestrial ecosystems. Nutrient recycling is a supporting service that controls the movement and exchange of matter back into the production of biomass.
13		**Available N**	Soil available nitrogen (mg N kg^{-1} soil)	Replicate-level	
14	**C cycling**	**Soil C stocks**	Soil carbon stocks (kg C m^{-2})	Replicate-level	Soils contain more than twice the amount of C stored in vegetation globally. Soil C storage and respiration rates are essential ecosystem functions that regulate the amount of CO_2 in the atmosphere, and therefore the Earth's climate. C-source degradation and extracellular enzyme activities are provided by soil bacteria, fungi, and archaea and are involved in the decomposition and mineralization of soil organic matter from complex polymers to simpler monomers. Enzyme activities and C degradation functions contribute to regulating the amount of litter and organic matter in our ecosystems.
15		**Soil respiration**	Soil respiration (µg CO_2-C g^{-1} soil day^{-1})	Plot-level	
16		**Sugar degradation**	Activity of beta-glucosidase (nmol activity g^{-1} dry soil hr^{-1})	Replicate-level	
17		**Chitin degradation**	Activity of N-acetylglucosaminidase (nmol activity g^{-1} dry soil hr^{-1})	Replicate-level	
18		**P mineralization**	Activity of phosphatase (nmol activity g^{-1} dry soil hr^{-1})	Replicate-level	
19		**Lignin degradation**	Lignin induced respiration (µg CO_2-C g^{-1} h^{-1})	Replicate-level	
20		**Glucose respiration**	Glucose respiration (µg CO_2-C g^{-1} soil day^{-1})	Replicate-level	

**21**	**Plant production**	**Plant productivity**	NDVI index (250m resolution)	Plot-level	Plant production is a key ecosystem process that supports important provisioning services such as food and fiber production, and that maintains ecosystem functioning via litter inputs. The proportion of woody plants present provides important information on the availability of timber resources, which are critical materials for building and construction needs.
**22**		**Wood resources**	Relative abundance of woody plants (%)	Plot-level	
**23**	**Vegetation composition**	**Proportion of forbs**	Proportion of forbs (%)	Transect-level	The community composition (%) of major functional groups of plants including forbs, grasses, shrubs and trees, provides essential structural information on the within-plot organization of vegetation. Vegetation structure is a major regulator of ecosystem functioning and often changes greatly during ecosystem development.
**24**		**Proportion of grasses**	Proportion of grasses (%)	Transect-level	
**25**		**Proportion of shrubs**	Proportion of shrubs (%)	Transect-level	
**26**		**Proportion of trees**	Proportion of trees (%)	Transect-level	
**27**	**Microbial structure**	**Total microbial biomass**	Microbial PLFAs content (nmol PLFA g^{-1} dry soil)	Replicate-level	Fungal and bacterial biomass and ratios provide structural information on the dominant type of microbes in soil food webs. Microbial biomass is a major controller of ecosystem functioning. The relative abundance of mycorrhizal fungi provides information on the potential capacity of soil to establish symbiosis with plants.
**28**		**Bacterial biomass**	Bacterial PLFAs content (nmol PLFA g^{-1} dry soil)	Replicate-level	
**29**		**Fungal biomass**	Fungal PLFAs content (nmol PLFA g^{-1} dry soil)	Replicate-level	
**30**		**Fungal: bacterial ratio**	Fungal: bacterial PLFAs ratio	Replicate-level	
**31**		**Ectomycorrhizal fungi**	Soil ectomycorrhizal fungi (%)	Replicate-level	
**32**		**Arbuscular mycorrhizal fungi**	Arbuscular mycorrhizal fungi (%)	Replicate-level	

Supplementary Table 4 | State factors of ecosystem development included in this study.

#	Group of variables	Variable	Units	Rationale
**1**	**Soil age**	Quantitative soil age	Estimated soil age in years (log-transformed)	Soil age is expected to be a fundamental predictor of the changes in ecosystem structure and function over time.
**2**		Semi-quantitative age	Standardized soil age (0-1) based on chronosequence stage calculated for each chronosequence independently	
**3**		Categorical soil age	Thousands of years = 1, hundreds of thousands of years = 2, millions of years = 3	
**4**	**Climate**	Maximum temperature	ºC	Climate is one of the major controllers of the changes in ecosystem function and structure over time. Temperature and precipitation control the rates of biological and chemical activity and thus regulates the rate of ecosystem development. Soils can develop faster in wetter and warmer climates.
**5**		Minimum temperature	ºC	
**6**		Temperature seasonality	standard deviation *100	
**7**		Mean diurnal temperature range	ºC	
**8**		Mean annual precipitation	mm	
**9**		Precipitation seasonality	Coefficient of Variation, %	
**10**		Climatic biome type	Biome (0 = mesic, 1 = dryland). Drylands have an Aridity Index < 0.65 and Mesic have an Aridity Index > 0.65, where the Aridity Index = Precipitation / Potential Evapotranspiration	
**11**	**Vegetation type**	Coniferous forests	0/1	Vegetation type represents one of the five major state factors that regulate soil and ecosystem development (i.e., organisms). Different types of vegetation types (forest, grasslands or shrublands) have different capacities to regulate soil erosion, accumulate plant litter and humus and influence microbial activity.
**12**		Angiosperm forests	0/1	
**13**		Grassland	0/1	
**14**		Shrubland	0/1	
**15**	**Parent material**	Chronosequence substrate: Sand dune	0/1	Parent material is a fundamental driver of ecosystem structure and function because it is the key point of origin of many mineral nutrients and because it affects soil properties such as texture and pH, and influences soil formation rates.
**16**		Chronosequence substrate: Volcanic	0/1	
**17**		Chronosequence substrate: Sedimentary	0/1	
**18**		Lithology: Basic volcanic rocks	0/1	
**19**		Lithology: Siliciclastic sedimentary rocks	0/1	
**20**		Lithology: Metamorphic rocks	0/1	
**21**		Lithology: Carbonate sedimentary rocks	0/1	
**22**		Lithology: Evaporites	0/1	
**23**		USDA soil class: Orthods	0/1	
**24**		USDA soil class: Orthents	0/1	
**25**		USDA soil class: Ustolls	0/1	
**26**		USDA soil class: Xerolls	0/1	
**27**		USDA soil class: Ustox	0/1	
**28**	**Topography**	Elevation	mm	Topography influences exposure to climate, fire, and other natural and anthropogenic factors, and regulates important ecosystem properties such as nutrient cycling, C stocks, plant productivity and soil properties.
**29**		Slope	Degrees from horizontal	
**30**		Aspect class	Four topographic orientation classes based on their levels of exposure to solar radiation*.<br>**Class 1:** Lowest levels of radiation (343-359º and 0-62º in the Northern Hemisphere, and 153-252º in the Southern Hemisphere)<br>**Class 2:** Low-medium levels of radiation (63-116º and 298-342º in the Northern Hemisphere, and 117-152º and 253-297º in the Southern Hemisphere)<br>**Class 3:** Medium-high levels of radiation (117-152º and 253-297º in the Northern Hemisphere, and 63-116º and 298-342º in the Southern Hemisphere)	

	**Class 4:** Highest levels of radiation (153-252º in the Northern Hemisphere, and 343-359º and 0-62º in the Southern Hemisphere)

*Parker, A.J. (1982) The topographic relative moisture index: An approach to soil-moisture assessment in mountain terrain. *Physical Geography* 3, 160-168.

Supplementary Table 5 | P-values arising from Variation Partitioning modeling in Fig. 1. See Supplementary Table 4 for a list of predictors and a rationale on their importance. See Supplementary Table 3 for a list of ecosystem structural and functional properties and a rationale for their significance.

Ecosystem property	n	Soil age	Climate	Vegetation	Parent material	Topography
Chemical Index of Alteration	435	<0.001	<0.001	<0.001	<0.001	<0.001
Total base cation reserve	435	<0.001	<0.001	<0.001	<0.001	<0.001
Fine texture	87	0.015	<0.001	0.025	<0.001	0.102
Soil pH	435	<0.001	<0.001	<0.001	<0.001	<0.001
Soil total P -H2SO4	435	<0.001	<0.001	<0.001	<0.001	<0.001
Soil total P -HF	435	0.043	<0.001	<0.001	<0.001	<0.001
Soil C:P ratio	435	<0.001	<0.001	<0.001	<0.001	<0.001
Soil N:P ratio	435	<0.001	<0.001	<0.001	<0.001	<0.001
Soil C:N ratio	435	0.002	<0.001	<0.001	<0.001	<0.001
Potential infiltration	435	<0.001	<0.001	<0.001	<0.001	<0.001
Water holding capacity	435	0.031	<0.001	<0.001	<0.001	<0.001
Available P	435	<0.001	<0.001	<0.001	<0.001	<0.001
Available N	435	0.002	<0.001	<0.001	<0.001	0.108
Soil C stocks	435	<0.001	<0.001	<0.001	<0.001	<0.001
Soil respiration	87	0.198	0.008	0.004	0.020	0.195
Sugar degradation	435	<0.001	<0.001	<0.001	<0.001	<0.001
Chitin degradation	435	<0.001	<0.001	0.033	<0.001	<0.001
P mineralization	435	<0.001	<0.001	<0.001	<0.001	<0.001
Lignin degradation	435	0.145	0.002	0.061	0.011	0.004
Glucose respiration	87	0.923	<0.001	<0.001	<0.001	0.047
Plant productivity	435	0.007	<0.001	<0.001	<0.001	<0.001
Wood resources	87	0.342	<0.001	<0.001	<0.001	0.303
Proportion of forbs	261	0.074	<0.001	<0.001	<0.001	0.605
Proportion of grasses	261	<0.001	<0.001	<0.001	<0.001	0.01
Proportion of shrubs	261	0.689	<0.001	<0.001	<0.001	<0.001
Proportion of trees	261	0.186	<0.001	<0.001	<0.001	<0.001
Total microbial biomass	435	<0.001	<0.001	<0.001	<0.001	0.008
Biomass bacteria	435	<0.001	<0.001	<0.001	<0.001	<0.001
Biomass fungi	435	<0.001	<0.001	<0.001	<0.001	0.015
Fungi: bacteria ratio	435	0.006	<0.001	<0.001	<0.001	0.006
Arbuscular mycorrhizal fungi	435	0.551	<0.001	<0.001	<0.001	<0.001
Ectomycorrhizal fungi	435	0.809	<0.001	<0.001	<0.001	0.139

Supplementary Table 6 | Selected relationships between quantitative soil age and ecosystem properties across major biome categories (drylands vs. mesic) and substrate types. P values were derived from ordinary least square regressions.

Selected relationships	Ecosystem factor	n	R^2	Slope	P-value
Quantitative soil age - Soil pH	Drylands	200	0.298	-0.436	<0.001
	Mesic	235	0.478	-0.587	<0.001
Quantitative soil age - TRB	Drylands	200	0.158	-126.699	<0.001
	Mesic	235	0.055	-46.475	<0.001
Quantitative soil age - NPP	Drylands	40	0.161	0.051	0.010
	Mesic	47	0.444	0.114	<0.001
Quantitative soil age - Proportion of trees	Drylands	120	0.142	4.881	0.000
	Mesic	141	0.037	4.521	0.022
Quantitative soil age - CIA	Sand dune substrate	110	0.020	1.318	0.140
	Other substrates	325	0.046	2.579	<0.001
Quantitative soil age - Soil total P-HF	Sand dune substrate	110	0.156	-21.226	<0.001
	Other substrates	325	0.009	-54.585	0.081
Quantitative soil age - Available P	Sand dune substrate	110	0.057	-0.632	0.012
	Other substrates	325	0.121	4.383	<0.001
Quantitative soil age - Soil N:P	Sand dune substrate	110	0.606	10.982	<0.001
	Other substrates	325	0.122	1.333	<0.001
Quantitative soil age - Soil C stocks	Sand dune substrate	110	0.335	0.263	<0.001
	Other substrates	325	0.107	0.690	<0.001
Quantitative soil age - Total microbial biomass	Sand dune substrate	110	0.053	11.962	0.016
	Other substrates	325	0.051	278.146	<0.001
Quantitative soil age - Soil respiration	Sand dune substrate	22	0.005	-0.023	0.745
	Other substrates	65	0.047	0.944	0.082
Quantitative soil age - Soil C:P	Sand dune substrate	110	0.057	20.324	<0.001
	Other substrates	325	0.530	331.578	<0.001

Supplementary Table 7 | Spearman correlation values ($P < 0.05$; N in Supplementary Table 3) associated with Fig. 10. See Fig. 1 for the location and acronyms of these chronosequences.

Ecosystem properties	JOR	BOS	BOV	AZ	CO	MI	ICE	ALPS	CAL	WA	CI	CH	TA	MEX	QL	HA
**Chemical Index of Alteration**		0.442	0.714	0.481	0.581					0.770		-0.405		0.400	-0.427	0.434
**Total base cation reserve**				-0.737	-0.617			-0.604		-0.849						
**Fine texture**				1.000	0.829	0.721					0.943				-0.943	
**Soil pH**	0.761	-0.799				-0.724	0.424	-0.843	-0.420	-0.954		-0.664			-0.488	-0.450
**Soil total P -H2SO4**	-0.799	-0.535			0.412	-0.642	0.565		-0.612	-0.923		0.527	0.318		-0.706	
**Soil total P -HF**	-0.745	-0.558		-0.853		-0.532		-0.404	-0.545	-0.908	-0.851	0.572				
**Soil C:P ratio**	0.605	0.768		0.481		0.858	-0.416	0.828		0.980	0.628	0.644	0.496		0.630	0.605
**Soil N:P ratio**	0.714	0.659		0.682		0.662	0.467	0.902	0.533	0.978	0.515	0.768	0.450		0.684	0.651
**Soil C:N ratio**	-0.659		-0.768			0.746	-0.498	-0.592		0.788						
**Potential infiltration**	-0.536					-0.668		0.577	-0.538		0.382					
**Water holding capacity**				0.768			-0.640	0.745	-0.565					0.397	-0.421	
**Available P**		0.582	-0.481	0.682	0.432	0.378		0.843	-0.886	-0.871	0.826	0.560				0.465
**Available N**	0.768						0.839	0.522	-0.783		0.466	0.587	0.613	0.441		
**Soil C stocks**		0.605		0.551		0.571		0.741		0.385	0.601	0.628	0.574	0.426		
**Soil respiration**				1.000							0.714					
**Sugar degradation**		0.648							0.463		0.723	0.416	0.465			
**Chitin degradation**			0.535						0.486	0.608	0.754	0.472				
**P mineralization**	-0.667	0.675	0.434	0.450		0.623				0.567	0.651	0.490				
**Lignin degradation**												-0.382				
**Glucose respiration**													-1.000			
**Plant productivity**	-1.000	0.949			-1.000	0.900	0.900	1.000	-0.900		0.886					
**Wood production**									-0.894			-0.928				
**Proportion of forbs**							0.659					0.857				
**Proportion of grasses**	-0.583					-0.730			0.914			0.573				
**Proportion of shrubs**				-0.637			0.761		-0.891							
**Proportion of trees**				0.777		0.493		0.702		0.702	0.663	0.512		0.572		
**Total microbial biomass**		0.698		0.636		0.606	0.620	0.832	0.384		0.739	0.605	0.768	0.502		
**Biomass bacteria**		0.714		0.690		0.615	0.643	0.828			0.739	0.628	0.752	0.577		
**Biomass fungi**		0.682		0.434		0.360		0.573	0.639	0.445	0.842	0.554	0.822			0.481
**Fungi: bacteria ratio**								-0.820	0.592	0.393	0.558	-0.793		-0.668	0.560	0.473
**Arbuscular mycorrhizal fungi**												0.673				0.458
**Ectomycorrhizal fungi**	0.456				-0.613	-0.379				-0.395		-0.371				

Supplementary Methods 1 | List of papers used to obtain information for our meta-analytical approach.

1. He, Y., Widney, S., Ruan, M., Herbert, E., Li, X., Craft, C. (2016) Accumulation of soil carbon drives denitrification potential and lab incubated gas production along a chronosequence of salt marsh development. Estuarine, Coastal and Shelf Science 172, 72-80.

2. Tarlera, S., Jangid, K., Ivester, A.H., Whitman, W.B., Williams, M.A. (2008). Microbial community succession and bacterial diversity in soils during 77000 years of ecosystem development. FEMS Microbiol Ecol 64, 129–140.

3. Frenot, Y., Van Vliet-Lanoë, B., Gloaguen, J-C. (1995). Particle Translocation and Initial Soil Development on a Glacier Foreland, Kerguelen Islands, Subantarctic. Arctic and Alpine Research, 27, 107-115.

4. Yoshitake, S., Uchida, M., Nakatsubo, T., Kanda, H. (2006). Characterization of soil microflora on a successional glacier foreland in the high Arctic on Ellesmere Island, Nunavut, Canada using phospholipid fatty acid analysis. Polar Biosci., 19, 73-84.

5. Kao-Kniffin, J., Woodcroft, B.J., Carver, S.M., Bockheim, J.G., Handelsman, J., Tyson, G.W., Hinkel, K.M., Mueller, C.W. (2015). Archaeal and bacterial communities across a chronosequence of drained lake basins in Arctic Alaska. Sci Rep. 5, 18165.

6. Zona, D. *et al.* (2010). Characterization of the carbon fluxes of a vegetated drained lake basin chronosequence on the Alaskan Arctic Coastal Plain. Glob. Change Biol. 16, 1870–1882.

7. Blaško, R. *et al.* (2015). Shifts in soil microbial community structure, nitrogen cycling and the concomitant declining N availability in ageing primary boreal forest ecosystems. Soil Biology and Biochemistry 91, 200-211.

8. Vilmundardóttir, O.K. *et al.* (2015). Between ice and ocean; soil development along an age chronosequence formed by the retreating Breiðamerkurjökull glacier, SE-Iceland. Geoderma 259–260.

9. Ibáñez, J-J-, Effland, W.R. (2011) Toward a Theory of Island Pedogeography: Testing the driving forces for pedological assemblages in archipelagos of different origins. Geomorphology 135, 215–223.

10. Bockheim, J G, Langley-Turnbaugh, S. (1997). Biogeochemical cycling in coniferous ecosystems on different aged marine terraces in coastal Oregon. Journal of Environmental Quality 26, 292.

11. Chen, C., *et al.* (2015). Soil phosphorus fractionation and nutrient dynamics along the Cooloola coastal dune chronosequence, southern Queensland, Australia. Geoderma, 257-258, 4–13.

12. Wardle D.A. *et al.* (2004). Ecosystem Properties and Forest Decline in Contrasting Long-Term Chronosequences. Science 305, 509.

13. Wardle. D.A. *et al.* (2009). Among- and within-species variation in plant litter decomposition in contrasting long-term chronosequences. Functional Ecology, 23, 442–453.

14. Wardle. D.A. *et al.* (2008). The response of plant diversity to ecosystem retrogression: evidence from contrasting long-term chronosequences. Oikos 117, 93-103.

15. Skelenar, P. *et al.* (2010). Primary succession of high-altitude Andean vegetation on lahars of Volcán Cotopaxi, Ecuador. Phytocoenologia 40, 15–28.

16. Lawrence C.R. *et al.* (2015) Long-term controls on soil organic carbon with depth and time: A casestudy from the Cowlitz River Chronosequence, WA USA. Geoderma 247–248, 73–87.

17. Dethier, D.P. (1988). The soil chronosequence along the Cowlitz River, Washington (U.S. Geological Survey Bulletin 1590-B).

18. Singleto, G.A., Lavkulich, L.M. (1987) Phosphorus transformations in a soil chronosequence, Vancouver Island, British Columbia. Can J Soil Sci 67:787–793.

19. Cornelis, J-T. *et al.* (2014) Silicon isotopes record dissolution and re-precipitation of pedogenic clay minerals in a podzolic soil chronosequence. Geoderma 235–236, 19–29.

20. Noll, M., Wellinger, N. (2008) Changes of the soil ecosystem along a receding glacier: Testing the correlation between environmental factors and bacterial community structure. Soil Biology and Biochemistry 40 2611–2619.

21. Wiederhold, J.G. *et al.* (2011) Chemical and biological gradients along the damma glacier soil chronosequence, switzerland. Vadose Zone Journal 10, 867-883.

22. Dumig, A. *et al.* (2012) Clay fractions from a soil chronosequence after glacier retreat reveal the initial evolution of organo–mineral associations. Geochimica et Cosmochimica Acta 85, 1–18.

23. Smittenberg, R.H.*et al.* (2012). Climate-sensitive ecosystem carbon dynamics along the soil chronosequence of the Damma glacier forefield, Switzerland. Global Change Biology 18, 1941–1955.

24. Dumig, A. *et al.* (2011) Concurrent evolution of organic and mineral components during initial soil development after retreat of the Damma glacier, Switzerland. Geoderma 163, 83–94.

25. Guelland K. *et al.* (2013). Mineralisation and leaching of C from 13C labelled plant litter along an initial soil chronosequence of a glacier forefield. Soil Biology and Biochemistry, 57 237-247.

26. Hans Göransson *et al.* (2016). Nitrogen and phosphorus availability at early stages of soil development in the Damma glacier forefield, Switzerland; implications for establishment of N2-fixing plants. Plant Soil 404, 251.

27. Brankatsch, R. *et al.* (2011). Abundances and potential activities of nitrogen cycling microbial communities along a chronosequence of a glacier forefield. The ISME Journal (2011) 5, 1025–1037.

28. Welc, M. *et al.* (2012). Soil bacterial and fungal communities along a soil chronosequence assessed by fatty acid profiling. Soil Biology and Biochemistry 49, 184-192.

29. Hagedorn, F. *et al.* (2015). Tracking litter-derived dissolved organic matter along a soil chronosequence using 14C imaging: Biodegradation, physico-chemical retention or preferential flow?. Soil Biology and Biochemistry 88, 333-343

30. Prietzel, J. *et al.* (2013). Soil sulphur speciation in two glacier forefield soil chronosequences assessed by S K-edge XANES spectroscopy. European Journal of Soil Science, April 2013, 64, 260–272

31. Guelland, K. *et al.* (2013) Evolution of carbon fluxes during initial soil formation along the forefield of Damma glacier, Switzerland. Biogeochemistry 113, 545–561.

32. Welc, M. *et al.* (2014). Rhizosphere fungal assemblages and soil enzymatic activities in a 110-years alpine chronosequence. Soil Biology & Biochemistry 74, 21-30.

33. Göransson, H. *et al.* (2011). Soil bacterial growth and nutrient limitation along a chronosequence from a glacier forefield. Soil Biology & Biochemistry 43, 1333-1340.

34. Frey, B. *et al.* (2013). Molecular characterization of phototrophic microorganisms in the forefield of a receding glacier in the Swiss Alps. Environ. Res. Lett. 8, 015033.

35. Tamburini, F. *et al.* (2012) Oxygen Isotopes Unravel the Role of Microorganisms in Phosphate Cycling in Soils. Environ. Sci. Technol. 46, 5956−5962.

36. Esperschutz, J. *et al.* (2011). Microbial food web dynamics along a soil chronosequence of a glacier forefield. Biogeosciences, 8, 3283–3294.

37. Graf, M. *et al.* (2007) Geochemical fractions of copper in soil chronosequences of selected European floodplains. Environmental Pollution 148, 788-796.

38. Todorovic, G.R. *et al.* (2014). Modeling and prediction of C dynamics in soil chronosequences of the critical zone observatory (CZO) Marchfeld/Austria. Catena 121, 53–67.

39. Zehetner, F. *et al.* (2009). Rates of biogeochemical phosphorus and copper redistribution in young floodplain soils. Biogeosciences 6, 2949–2956.

40. Zehetner, F. *et al.* (2008). From sediment to soil: floodplain phosphorus transformations at the Danube River. Biogeochemistry 88, 117–126.

41. Lair, G.J. *et al.* (2009). Phosphorus sorption–desorption in alluvial soils of a young weathering sequence at the Danube River. Geoderma 149, 39–44

42. Liu, G-X. *et al.* (2012) Variations in soil culturable bacteria communities and biochemical characteristics in the Dongkemadi glacier forefield along a chronosequence. Folia Microbiol 57, 485–494.

43. Turner, B.L. *et al.* (2016) Sulfur dynamics during long-term ecosystem development. Biogeochemistry 128, 281.

44. Laliberte, E. *et al.* (2013) How does pedogenesis drive plant diversity? Trends in Ecology & Evolution 28, 6.

45. Richardson, S.J. *et al.* (2004). Rapid development of phosphorus limitation in temperate rainforest along the Franz Josef soil chronosequence. Oecologia 139, 267–276.

46. Menge, D.N., Hedin, L.O. (2009). Nitrogen fixation in different biogeochemical niches along a 120 000-year chronosequence in New Zealand. Ecology 90, 2190–2201.

47. Menge, D.N.L. *et al.* (2011). Declining foliar and litter d15N diverge from soil, epiphyte and input d15N along a 120 000 yr temperate rainforest chronosequence. New Phytologist 190, 941–952.

48. Turner, B.L. *et al.* (2013) Soil microbial biomass and the fate of phosphorus during long-term ecosystem development. Plant Soil 367, 225–234.

49. Mason, N.W.H. *et al.* (2012) Changes in coexistence mechanisms along a long-term soil chronosequence revealed by functional trait diversity. Journal of Ecology 100, 678–689.

50. Allison, V.J. *et al.* (2007) Changes in enzyme activities and soil microbial community composition along carbon and nutrient gradients at the Franz Josef chronosequence, New Zealand. Soil Biology and Biochemistry 39, 1770–1781.

51. Enrique Doblas-Mirandaa, E. *et al.* (2008). Changes in the community structure and diversity of soil invertebrates across the Franz Josef Glacier chronosequence. Soil Biology and Biochemistry 40, 1069–1081.

52. Kornfeld, A. *et al.* (2013) Modulation of respiratory metabolism in response to nutrient changes along a soil chronosequence. Plant, Cell and Environment 36, 1120–1134.

53. Turner, S. *et al.* (2014) Mineralogical impact on long-term patterns of soil nitrogen and phosphorus enzyme activities. Soil Biology and Biochemistry 68, 31-43.

54. Parfitt, R.L. *et al.* (2005) N and P in New Zealand soil chronosequences and relationships with foliar N and P. Biogeochemistry 75, 305–328.

55. Holdaway, R.J. *et al.* (2011) Species- and community-level patterns in fine root traits along a 120 000-year soil chronosequence in temperate rain forest. Journal of Ecology 99, 954–963.

56. Turner, B.L. *et al.* (2007) Soil Organic Phosphorus Transformations During Pedogenesis. Ecosystems 10, 1166–1181.

57. Garibotti, I.A. *et al.* (2011). Vegetation Development on Deglaciated Rock Outcrops from Glaciar Frías, Argentina. Arctic, Antarctic, and Alpine Research 43, 35-45.

58. Bormann, B.T., Sidle, R.C. (1990) Changes in Productivity and Distribution of Nutrients in a Chronosequence at Glacier BayNational Park, Alaska. Journal of Ecology 78, 561-578.

59. Egli, M. (2012) Soil organic carbon and nitrogen accumulation rates in cold and alpine environments over 1 Ma. Geoderma 183-184, 109–123.

60. Birkeland, P.W. *et al.* (1987) Holocene Alpine Soils in Gneissic Cirque Deposits, Colorado Front Range (U.S. Geological Survey Bulletin 1590-E).

61. Sauer, D. *et al.* (2007) A soil chronosequence in the semi-arid environment of Patagonia (Argentina). Catena 71, 382–393.

62. Roberts, K. *et al.* (2015) Oxygen isotopes of phosphate and soil phosphorus cycling across a 6500 year chronosequence under lowland temperate rainforest. Geoderma 257–258, 14–21.

63. Eger, A. *et al.* (2011). Pedogenesis, soil mass balance, phosphorus dynamics and vegetation communities across a Holocene soil chronosequence in a super-humid climate, South Westland, New Zealand. Geoderma 163, 185–196.

64. Turner, B.L. *et al.* (2014) Soil organic phosphorus transformations along a coastal dune chronosequence under New Zealand temperate rain forest. Biogeochemistry 121:595–611.

65. Jangid, K. *et al.* (2013). Progressive and retrogressive ecosystem development coincide with soil bacterial community change in a dune system under lowland temperate rainforest in New Zealand. Plant Soil 367, 235–247.

66. Turner, B.L. *et al.* (2012). Soil nutrient dynamics during podzol development under lowland temperate rain forest in New Zealand. Catena 97, 50–62.

67. Benjamin L. Turner, B.L. *et al.* (2012) Patterns of tree community composition along a coastal dune chronosequence in lowland temperate rain forest in New Zealand. Plant Ecol 213, 1525–1541.

68. Wang, J. *et al.* (2016) Carbon demand drives microbial mineralization of organic phosphorus during the early stage of soil development. Biol Fertil Soils DOI 10.1007/s00374-016-1123-7.

69. Lei, Y. *et al.* (2015) Soil nematode assemblages as bioindicators of primary succession along a 120-year-old chronosequence on the Hailuogou Glacier forefield, SW China. Soil Biology and Biochemistry 88, 362-371.

70. Wu, Y. *et al.* (2015), Rapid loss of phosphorus during early pedogenesis along a glacier retreat choronosequence, Gongga Mountain (SW China). PeerJ 3:e1377; DOI 10.7717/peerj.1377.

71. Zhou, J. *et al.* (2013) Changes of soil phosphorus speciation along a 120-year soil chronosequence in the Hailuogou Glacier retreat area (Gongga Mountain, SW China). Geoderma 195–196, 251–259.

72. Zhou, J. *et al.* (2016) Rapid weathering processes of a 120-year-old chronosequence in the Hailuogou Glacier foreland, Mt. Gongga, SW China. Geoderma 267, 78–91.

73. Prietzel, J. *et al.* (2013) Synchrotron-based P K-edge XANES spectroscopy reveals rapid changes of phosphorus speciation in the topsoil of two glacier foreland chronosequences. Geochimica et Cosmochimica Acta 108, 154–171.

74. Li, J-W. *et al.* (2013) Nd isotope evidence for dust accretion to a soil chronosequence in Hainan Island. Catena 101, 24–30.

75. Huang, L-M. *et al.* (2016) Pedogenesis significantly decreases the stability of water-dispersible soil colloids in a humid tropical region. Geoderma 274, 45–53.

76. Gillespie, R.G. *et al.* (2016) Island time and the interplay between ecology and evolution in species diversification. Evolutionary Applications 9, 53–73.

77. Crews, T.E. *et al.* (1995) Changes in Soil Phosphorus Fractions and Ecosystem Dynamics across a Long Chronosequence in Hawaii. Ecology 76, 1407–1424.

78. Olander, L.P., Vitousek, P.M. (2000) Regulation of soil phosphatase and chitinase activity by N and P availability. Biogeochemistry 49, 175–190.

79. Hobbie, S.E., Vitousek, P.M. (2000) Nutrient limitation of decomposition in Hawaiian forests. Ecology 81: 1867–1877.

80. Rillig, M.C. *et al.* (2001) Large contribution of arbuscular mycorrhizal fungi to soil carbon pools in tropical forest soils. Plant and Soil 233, 167–177.

81. Reed. S.C. *et al.* (2011) Are patterns in nutrient limitation belowground consistent with those aboveground: results from a 4 million year chronosequence. Biogeochemistry 106, 323–336.

82. Crews, T.E. *et al.* (2000) Changes in Asymbiotic, Heterotrophic Nitrogen Fixation on Leaf Litter of Metrosideros polymorpha with Long-Term Ecosystem Development in Hawaii. Ecosystems 3, 386–395.

83. Ostertag, R., Hobbie, S.E. (1999) Early stages of root and leaf decomposition in Hawaiian forests: effects of nutrient availability. Oecologia 121, 564–573.

84. Gruner, D.S. *et al.* (2007) Geological age, ecosystem development, and local resource constraints on arthropod community structure in the Hawaiian Islands. Biological Journal of the Linnean Society 90, 551–570.

85. Kitayama, K. *et al.* (1997) Fate of a Wet Montane Forest During Soil Ageing in Hawaii. Journal of Ecology 85, 669-679.

86. Mikutta, R. *et al.* (2010) Mineralogical impact on organic nitrogen across a long-term soil chronosequence (0.3–4100 kyr). Geochimica et Cosmochimica Acta 74, 2142–2164.

87. Balser, T.C. *et al.* (2005) Using lipid analysis and hyphal length to quantify AM and saprotrophic fungal abundance along a soil chronosequence. Soil Biology and Biochemistry 37, 601–604.

88. Ostergard, R. (2001) Effects of nitrogen and phosphorus availability on fine-root dynamics in Hawaiian montane forest. Ecology 82, 485–499.

89. Herbert, D.A., Fownes, J.H. (1999) Forest Productivity and Efficiency of Resource Use Across a Chronosequence of Tropical Montane Soils. Ecosystems 2, 242–254.

90. Kitayama, K. (1996) Soil nitrogen dynamics along a gradient of long-term soil development in a Hawaiian wet montane rainforest. Plant and Soil 183, 253-262.

91. Crews, T.E. *et al.* (2001) Organic matter and nitrogen accumulation and nitrogen fixation during early ecosystem development in Hawaii. Biogeochemistry 52, 259–279.

92. Vitousek, P.M. *et al.* (2009) Landscape-level variation in forest structure and biogeochemistry across a substrate age gradient in Hawaii. Ecology 90, 3074–3086.

93. Saldaña, A. *et al.* (1998). Spatial variability of soil properties at different scales within three terraces of the Henares River (Spain). Catena 33, 139–153.

94. Dilustro, J.J., Day, F.P. (1997) Aboveground Biomass and Net Primary Production along a Virginia Barrier Island DuneChronosequence. The American Midland Naturalist, 137, 27-38.

95. Day, F.P. (1996) Effects of Nitrogen Availability on Plant Biomass along a Barrier Island DuneChronosequence. Castanea 61, 369-381.

96. Even, S.T. *et al.* (2010). Shrub expansion stimulates soil C and N storage along a coastal soil chronosequence. Global Change Biology 16, 2052–2061.

97. Stevenson, M.J. (1995). Fine root biomass distribution and production along a barrier island chronosequence. P.h.d. thesis. Old Dominion University, Virginia, USA.

98. Conn, C.E., Day, F.P. (1997) Root decomposition across a barrier island chronosequence: Litter quality and environmental controls. Plant and Soil 195, 351–364.

99. Tyler, C.A. *et al.* (2003) Nitrogen fixation and nitrogen limitation of primary production along a natural marsh chronosequence. Oecologia 136, 431–438.

100. Zemunik, G. *et al.* (2016) Increasing plant species diversity and extreme species turnover accompany declining soil fertility along a long-term chronosequence in a biodiversity hotspot. Journal of Ecology 104, 792–805.

101. Albornoz, F.E. *et al.* (2016) Shifts in symbiotic associations in plants capable of forming multiple root symbioses across a long-term soil chronosequence. Ecology and Evolution 6, 2368–2377.

102. Kruger, M. *et al.* (2015) The rise and fall of arbuscular mycorrhizal fungal diversity during ecosystem retrogression. Molecular Ecology 24, 4912–4930.

103. Laliberte, E. *et al.* (2012) Experimental assessment of nutrient limitation along a 2-million-year dune chronosequence in the south-western Australia biodiversity hotspot. Journal of Ecology 100, 631–642.

104. Teste, F.P. *et al.* (2016). Mycorrhizal fungal biomass and scavenging declines in phosphorusimpoverished soils during ecosystem retrogression. Soil Biology and Biochemistry 92, 119-132.

105. Hayes, P. *et al.* (2014) Foliar nutrient concentrations and resorption efficiency in plants of contrasting nutrient-acquisition strategies along a 2-million-year dune chronosequence. Journal of Ecology 102, 396–410.

106. Turner, B.L., Laliberte, E. (2015) Soil Development and Nutrient Availability Along a 2 Million-Year Coastal Dune Chronosequence Under Species-Rich Mediterranean Shrubland in Southwestern Australia. Ecosystems 18, 287–309.

107. Reheis, M.C. *et al.* (1987) Gypsic Soils on the Kane Alluvial Fans, Big Horn County, Wyoming (U.S. Geological Survey Bulletin 1590-C).

108. Carlson, M.L. *et al.* (2010) Community development along a proglacial chronosequence: are above-ground and below-ground community structure controlled more by biotic than abiotic factors? Journal of Ecology 98, 1084–1095.

109. Rhoades, C. *et al.* (2008) Soil nitrogen accretion along a floodplain terrace chronosequence in northwest Alaska: Influence of the nitrogen-fixing shrub Shepherdia canadensis. Ecoscience 15, 223-230.

110. Moon, J. *et al.* (2016) Plant e Microbial and mineral contributions to amino acid and protein organic matter accumulation during 4000 years of pedogenesis. Soil Biology and Biochemistry 100, 42-50.

111. Williams, M.A. *et al.* (2013) Bacterial communities in soil mimic patterns of vegetative succession and ecosystem climax but are resilient to change between seasons. Soil Biology and Biochemistry 57, 749-757.

112. Lichter, J. (1998) Primary Succession and Forest Development on Coastal Lake Michigan Sand Dunes. Ecological Monographs 68, 487-510.

113. Lajtha, K., Schlesinger, W.S. (1988) The Biogeochemistry of Phosphorus Cycling and Phosphorus Availability Along a Desert Soil Chronosequence. Ecology 69, 24-39.

114. Lajtha, K. *et al.* (1988). The use of ion-exchange resin bags for measuring nutrient availability in an arid ecosystem. Plant and Soil 105, 105-111.

115. Pérez, C.A. *et al.* (2014) Patterns of biological nitrogen fixation during 60 000 years of forest development on volcanic soils from south-central Chile. New Zealand Journal of Ecology 38, 2.

116. Gallardo, M-B. *et al.* (2012) Desacoplamiento del desarrollo del suelo y la sucesión vegetal a lo largo de una cronosecuencia de 60 mil años en el volcán Llaima, Chile. Revista Chilena de Historia Natural 85, 291-306.

117. Holliday, V.T. (1988). Genesis of a Late-Holocene Soil Chronosequence at the Lubbock Lake Archaeological Site, Texas. Annals of the Association of American Geographers, 78, 594-610.

118. Mouratov, S. *et al.* (2010) Seasonal Effect of Geomorphological Chronosequence Features on Soil Biota Dynamics. Pedosphere 20, 761–770.

119. Syers, J.K., Walker, T.W. (1969) Phosphorus transformations in a chronosequence of soils developed on wind-blown sand in New Zealand I. total and organic phosphorus. Journal of Soil Science 20, 1.

120. Syers, J.K., Walker, T.W. (1969) Phosphorus transformations in a chronosequence of soils developed on wind-blown sand in New Zealand II. Inorganic phosphorus. Journal of Soil Science 20, 3.

121. Mcdowell, R.W. *et al.* (2007) Organic phosphorus speciation and pedogenesis: analysis by solution 31P nuclear magnetic resonance spectroscopy. European Journal of Soil Science 58, 1348–1357.

122. Burt, R., Alexander, E.B. (1996) Soil development on moraines of Mendenhall Glacier, southeast Alaska. 2. Chemical transformations and soil micromorphology. Geoderma 72, 19-36.

123. Harden, J.W. *et al.* (1987) Soils Developed in Granitic Alluvium near Merced, California. (U.S. Geological Survey Bulletin 1590-A).

124. White, A.F. et al (2005) Chemical weathering rates of a soil chronosequence on granitic alluvium: III. Hydrochemical evolution and contemporary solute fluxes and rates. Geochimica et Cosmochimica Acta 69, 1975–1996.

125. Gracheva, R. (2011) Formation of soil diversity in the mountainous tropics and subtropics: Rocks, time, and erosion. Geomorphology 135, 224–231.

126. Pietrasiak, N. (2014) Biogeomorphology of a Mojave Desert landscape — Configurations and feedbacks of abiotic and biotic land surfaces during landform evolution. Geomorphology 206, 23–36.

127. Aanderud, Z.T. (2008) Shrub-interspace dynamics alter relationships between microbial community composition and belowground ecosystem characteristics. Soil Biology and Biochemistry 40, 2206–2216.

128. Reheis, M.C. (1987) Soils in Granitic Alluviumin Humid and Semiarid Climatesalong Rock Creek, Carbon County, Montana. (U.S. Geological Survey Bulletin 1590-D).

129. Eckmeier, E. *et al.* (2013) Black carbon contributes to organic matter in young soils in the Morteratsch proglacial area (Switzerland). Biogeosciences 10, 1265–1274.

130. Egli, M. *et al.* (2010) Soil organic matter formation along a chronosequence in the Morteratsch proglacial area (Upper Engadine, Switzerland). Catena, 82, 61-69.

131. Egli, M. (2012) Rapid transformation of inorganic to organic and plant-available phosphorous in soils of a glacier forefield. Geoderma 189–190, 215–226.

132. Albrecht, M. *et al.* (2010) Plant–pollinator network assembly along the chronosequence of a glacier foreland. Oikos 119: 1610–1624.

133. Cutler, N. (2011) Nutrient limitation during long-term ecosystem development inferred from a mat-forming moss. The Bryologist, 114, 204-214.

134. Cutler, N.A. *et al.* (2014) Long-term changes in soil microbial communities during primary succession. Soil Biology and Biochemistry 69, 359-370.

135. Dickson, B.A., Crocker, R.L. (1953) A chronosequence of soils and vegetation near Mt. Shasta, California. II the development of the forest floors and the carbon and nitrogen profiles of the soils. Journal of Soil Science 4, 2.

136. Lilienfein, J. *et al.* (2004) Adsorption of Dissolved Organic Carbon and Nitrogen in Soils of a Weathering Chronosequence. Soil Sci. Soc. Am. J. 68, 292–305.

137. Uselman, S.M. *et al.* (2007) Fine Root Production across a Primary Successional Ecosystem Chronosequence at Mt.Shasta, California. Ecosystems 10, 703-717.

138. Sollins, P. *et al.* (1983). Processes of Soil Organic-Matter Accretion at a Mudfloe Chronosequence, Mt. Shasta,California. Ecology 64, 1273-1282.

139. Lilienfein, J. *et al.* (2003). Soil formation and organic matter accretion in a young andesitic chronosequence at Mt. Shasta, California. Geoderma 116, 249–264.

140. Leinweber, P. *et al.* (1996). Molecular characterization of soil organic matter in Pleistocene moraines from the Bolivian Andes. Geoderma 72, 133-148.

141. Follmi, K.B. *et al.* (2009) Weathering and the mobility of phosphorus in the catchments and forefields of the Rhone and Oberaar glaciers, central Switzerland: Implications for the global phosphorus cycle on glacial–interglacial timescales. Geochimica et Cosmochimica Acta 73, 2252–2282.

142. Garnett, K.H. *et al.* (2011) Natural abundance radiocarbon in soil microbial biomass: Results from a glacial foreland. Soil Biology and Biochemistry 43, 1356-1361.

143. Bardgett, R.D. *et al.* (2007) Heterotrophic microbial communities use ancient carbon following glacial retreat. Biol. Lett. 3, 487–490.

144. Kandeler, E. *et al.* (2006) Abundance of narG, nirS, nirK, and nosZ Genes of Denitrifying Bacteria during Primary Successions of a Glacier Foreland. Applied and Environmental Microbiology 72, 5957–5962.

145. Hofmann, K. *et al.* (2013) Aerobic and anaerobic microbial activities in the foreland of a receding glacier. Soil Biology and Biochemistry 57, 418-426.

146. Tscherko, D. *et al.* (2004) Shifts in rhizosphere microbial communities and enzyme activity of Poa alpina across an alpine chronosequence. Soil Biology and Biochemistry 36, 1685–1698.

147. Kaufmann, R. *et al.* (2002) The Soil Fauna of an Alpine Glacier Foreland: Colonization and Succession. Arctic, Antarctic, and Alpine Research 34, 242-250.

148. Izquierdo, J.E. *et al.* (2013) Evidence for progressive phosphorus limitation over long-term ecosystem development: Examination of a biogeochemical paradigm. Plant Soil 367, 135–147.

149. Selmants, P.C., Hart, S.C (2008). Substrate age and tree islands influence carbon and nitrogen dynamics across a retrogressive semiarid chronosequence. Global Biogeochemical Cycles, 22. doi:10.1029/2007GB003062.

150. Selmants, P.C., Hart, S.C (2010). Phosphorus and soil development: Does the Walker and Syers model apply to semiarid ecosystems? Ecology Vol. 91, 474-484

151. Perez, C.A. *et al.* (2014) Ecosystem development in short-term postglacial chronosequences: N and P limitation in glacier forelands from Santa Inés Island, Magellan Strait. Austral Ecology 39, 288–303.

152. Wang, M. *et al.* (2016) Distribution of Root-Associated Bacterial Communities Along a Salt-Marsh Primary Succession. Front. Plant Sci.6:1188.

153. Dini-Andreote, F. *et al.* (2016) Reconstructing the GeneticPotential of the Microbially-Mediated Nitrogen Cycle in a Salt Marsh Ecosystem. Front. Microbiol.7:902.

154. Dini-Andreote, F. *et al.* (2014). Dynamics of bacterial community succession in a salt marsh chronosequence: evidences for temporal niche partitioning. The ISME Journal 8, 1989–2001

155. James, P. *et al.* (2016) Development and spatial distribution of soils on an active volcano: Mt Etna, Sicily. Catena 137, 277–297.

156. Peña-Ramírez, V.M. *et al.* (2009) Soil organic carbon stocks and forest productivity in volcanic ash soils of different age (1835–30,500 years B.P.) in Mexico. Geoderma 149, 224–234.

157. Reverchon, F. *et al.* (2010) Changes in community structure of ectomycorrhizal fungi associated with Pinus montezumae across a volcanic soil chronosequence at Sierra Chichinautzin, Mexico. Canadian Journal of Forest Research 40, 1161-1174.

158. Reverchon, F. *et al.* (2010) Saprophytic fungal communities change in diversity and species composition across a volcanic soil chronosequence at Sierra del Chichinautzin, Mexico. Annals of Microbiology 60, 217-226.

159. Galván-Tejada, N.C. *et al.* (2014) Soil P fractions in a volcanic soil chronosequence of Central Mexico and their relationship to foliar P in pine trees. J. Plant Nutr. Soil Sci. 177, 792–802.

160. Mahaney, W.C. *et al.* (2007) Soil stratigraphy and plant–soil interactions on a Late Glacial–Holocene fluvial terrace sequence, Sierra Nevada National Park, northern Venezuelan Andes. Journal of South American Earth Sciences 23, 46–60.

161. Vilmundardóttir, O.K. *et al.* (2014) Early stage development of selected soil properties along the proglacial moraines of Skaftafellsjökull glacier, SE-Iceland. Catena 121, 142–150.

162. Masiello, C.A. *et al.* (2004) Weathering controls on mechanisms of carbon storage in grassland soils. Global Biogeochemical Cycles 18, GB4023.

163. Darmody, R.G. *et al.* (2005) Soil Topochronosequences at Storbreen, Jotunheimen, Norway. Soil Science Society of America Journal 69, 1275.

164. Tsai, C-C. *et al.* (2007) Soil genesis along a chronosequence on marine terraces in eastern Taiwan Catena 71, 394–405.

165. Tsai, H. *et al.* (2016) Soilscape of west-central Taiwan: Its pedogenesis and geomorphic implications. Geomorphology 255, 81–94.

166. Gobbi, M. *et al.* (2006) Epigean Arthropod Succession along a 154-Year Glacier Foreland Chronosequence in theForni Valley (Central Italian Alps). Arctic, Antarctic, and Alpine Research 38, 357-362.

167. Wookey, P.A. *et al.* (2002) Surface Age, Ecosystem Development, and C Isotope Signatures of Respired CO2 in an Alpine Environment, North Iceland. Arctic, Antarctic, and Alpine Research, 34, 76-87.

168. Bate, D.B. *et al.* (2008) Soil phosphorus cycling in an Antarctic polar desert. Geoderma 144, 21–31.

169. Gracheva, R.G. *et al.* (2001) Time-dependent factors of soil and weathering mantle diversity in the humid tropics and subtropics: a concept of soil self-development and denudation. Quaternary International 78, 3-10.

170. Buckeridge, K.M. *et al.* (2016) Vegetation Leachate During Arctic Thaw Enhances Soil Microbial Phosphorus. Ecosystems 19, 477–489.

171. Whittinghill, K.A. *et al.* (2014) Bioavailability of dissolved organic carbon across a hillslope chronosequence in the Kuparuk River region, Alaska. Soil Biology and Biochemistry 79, 25-33.

172. Whittinghill, K.A., Hobbie, S.E. (2011) Effects of Landscape Age on Soil Organic Matter Processing in Northern Alaska. Soil Sci. Soc. Am. J. 75, 907–917.

173. Whittinghill, K.A., Hobbie, S.E. (2012) Effects of pH and calcium on soil organic matter dynamics in Alaskan tundra. Biogeochemistry 111, 569–581.

174. Vincent, A.G. *et al.* (2013) Soil organic phosphorus transformations in a boreal forest chronosequence. Plant Soil 367, 149–162

175. Harden, J.W. *et al.* (1986) Soils Developed on Coastal and Fluvial Terraces near Ventura, California. (U.S. Geological Survey Bulletin 1590-B).

176. Williamson, W.M. *et al.* (2005) Changes in soil microbial and nematode communities during ecosystem decline across a long-term chronosequence. Soil Biology and Biochemistry 37, 1289–1301.

177. Coomes, D.A. *et al.* (2013) Soil drainage and phosphorus depletion contribute to retrogressive succession along a New Zealand chronosequence. Plant Soil 367, 77–91.

178. Almond, P.C., Tonkin, P.J. (1999) Pedogenesis by upbuilding in an extreme leaching and weathering environment, and slow loess accretion, south Westland, New Zealand. Geoderma 92, 1–36.

179. Dahms, D. Egli, M. (2016) Carbonate and elemental accumulation rates in arid soils of mid-to-late Pleistocene outwash terraces, southeasternWind River Range, Wyoming, USA. Chemical Geology (in press).

180. Cline, L.C., Zak, D.R. (2014) Dispersal limitation structures fungal community assembly in a long-term glacial chronosequence. Environmental Microbiology 16, 1538–1548.

181. Freedman, Z., Zak, D.R. (2015) Soil bacterial communities are shaped by temporal and environmental filtering: evidence from a long-term chronosequence. Environmental Microbiology 17, 3208–3218

182. Bussaca, L.G. *et al.* (1989) Late Cenozoic stratigraphy of the Feather and Yuba rivers area, California, with a section on soil development in mixed alluvium at Honcut Creek. (U.S. Geological Survey Bulletin 1590-G).

Supplementary Methods 2 | PRISMA 2009 Flow Diagram associated with our meta-analysis.

PRISMA 2009 Flow Diagram

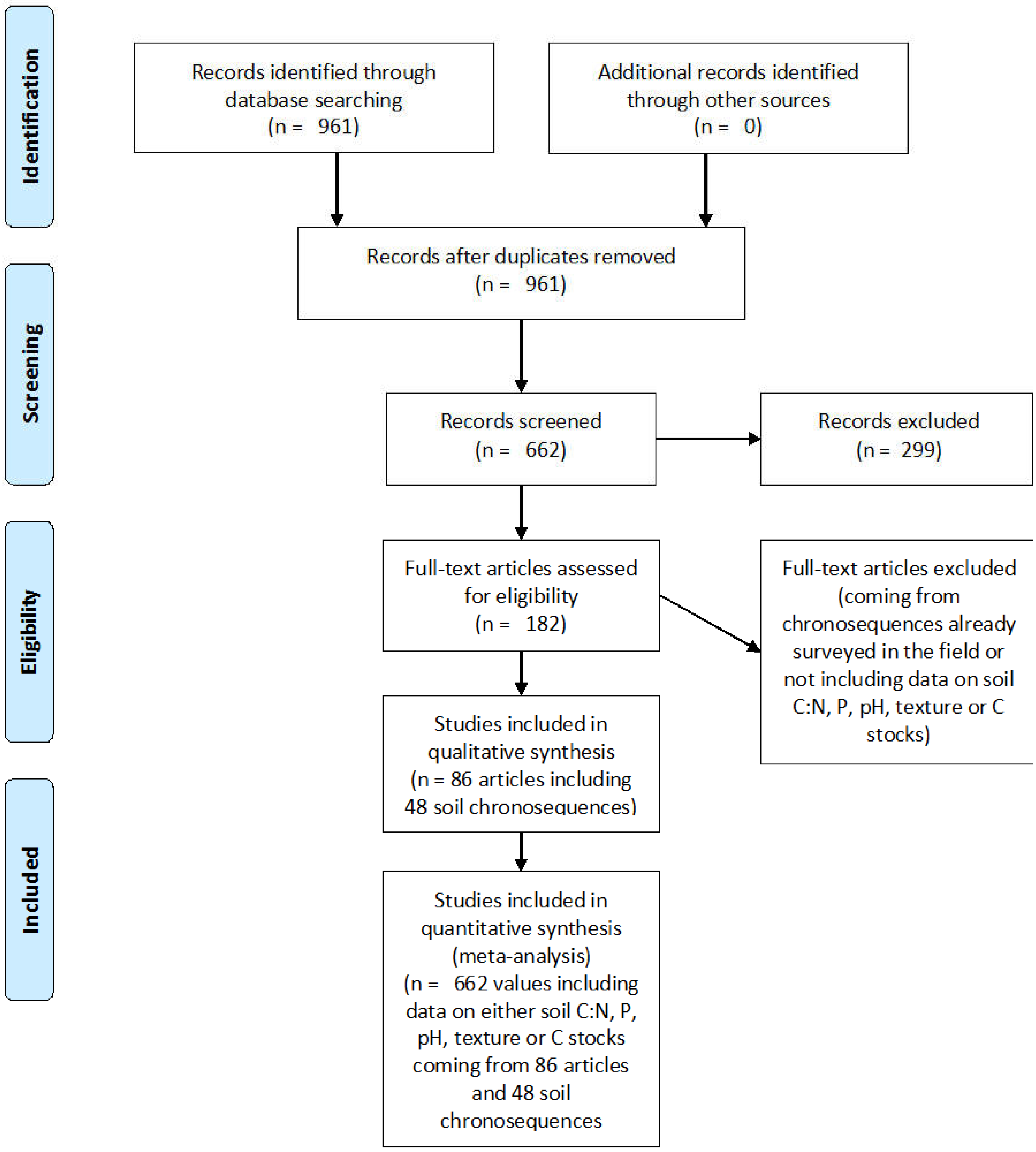

From: Moher D, Liberati A, Tetzlaff J, Altman DG, The PRISMA Group (2009). *P*referred *R*eporting *I*tems for *S*ystematic Reviews and *M*eta-*A*nalyses: The PRISMA Statement. PLoS Med 6(7): e1000097. doi:10.1371/journal.pmed1000097

For more information, visit www.prisma-statement.org.
