# Supplementary material for: The influence of soil age on ecosystem structure and function across biomes
Source: Nat Commun. 2020 Sep 18;11:4721. doi: 10.1038/s41467-020-18451-3 (PMC7501311; doi:10.1038/s41467-020-18451-3)
Supplement: Supplementary file 2 — Reporting Summary [file 41467_2020_18451_MOESM2_ESM.pdf]

nature research

Corresponding author(s): MANUEL DELGADO-BAQUERIZO

Last updated by author(s): Mar 19, 2020

Reporting Summary

Nature Research wishes to improve the reproducibility of the work that we publish. This form provides structure for consistency and transparency in reporting. For further information on Nature Research policies, see Authors & Referees and the Editorial Policy Checklist .

Statistics

For all statistical analyses, confirm that the following items are present in the figure legend, table legend, main text, or Methods section.

n/a	Confirmed	
☐	☒	The exact sample size (*n*) for each experimental group/condition, given as a discrete number and unit of measurement
☐	☒	A statement on whether measurements were taken from distinct samples or whether the same sample was measured repeatedly
☐	☒	The statistical test(s) used AND whether they are one- or two-sided<br>*Only common tests should be described solely by name; describe more complex techniques in the Methods section.*
☐	☒	A description of all covariates tested
☐	☒	A description of any assumptions or corrections, such as tests of normality and adjustment for multiple comparisons
☐	☒	A full description of the statistical parameters including central tendency (e.g. means) or other basic estimates (e.g. regression coefficient) AND variation (e.g. standard deviation) or associated estimates of uncertainty (e.g. confidence intervals)
☐	☒	For null hypothesis testing, the test statistic (e.g. *F*, *t*, *r*) with confidence intervals, effect sizes, degrees of freedom and *P* value noted<br>*Give P values as exact values whenever suitable.*
☒	☐	For Bayesian analysis, information on the choice of priors and Markov chain Monte Carlo settings
☒	☐	For hierarchical and complex designs, identification of the appropriate level for tests and full reporting of outcomes
☒	☐	Estimates of effect sizes (e.g. Cohen's *d*, Pearson's *r*), indicating how they were calculated

Our web collection on statistics for biologists contains articles on many of the points above.

Software and code

Policy information about availability of computer code

Data collection	We combined a new field survey including data for 33 soil, plant, and microbial properties and multifunctionality across 16 globally distributed soil chronosequences, with a global meta-analysis.
Data analysis	Statistical analyses were made with R 3.4.0. Used R packages, including the used codes, are clearly specified in the method section. References associated with the used bioinformatic pipelines are included in the method section.

For manuscripts utilizing custom algorithms or software that are central to the research but not yet described in published literature, software must be made available to editors/reviewers. We strongly encourage code deposition in a community repository (e.g. GitHub). See the Nature Research guidelines for submitting code & software for further information.

Data

Policy information about availability of data

All manuscripts must include a data availability statement. This statement should provide the following information, where applicable:

- Accession codes, unique identifiers, or web links for publicly available datasets
- A list of figures that have associated raw data
- A description of any restrictions on data availability

All the materials, raw data, and protocols used in the article are available upon request and without restriction, and all data will be made publicly available in ref. 52 (Figshare) upon publication.

Field-specific reporting

Please select the one below that is the best fit for your research. If you are not sure, read the appropriate sections before making your selection.

Ecological, evolutionary & environmental sciences study design

All studies must disclose on these points even when the disclosure is negative.

Study description	The importance of soil age as an ecosystem driver across global biomes remains largely undetermined. To fill this knowledge gap, here, we combined a new field survey including data for 32 soil, plant, and microbial properties across 16 globally distributed soil chronosequences, with a global meta-analysis.
Research sample	Vegetation information and soil samples coming from 16 globally distributed soil chronosequences. We complemented this information with a data synthesis from the literature including 48 additional comparable soil chronosequences. Detailed information on the investigated 16 chronosequences can be found in the method section, Fig. 1 and Supplementary Table 1. We also collected data from 48 global soil chronosequences. Information on these locations can be found in the method section, Fig. 3, Supplementary Table 2 and Supplementary Methods 1 and 2.
Sampling strategy	Soil and vegetation data were collected using standardized protocols between 2016 and 2017 from 16 soil chronosequences located in nine countries and six continents. Field surveys were conducted according to a standardized sampling protocol. We surveyed a 50 m × 50 m plot within each chronosequence stage, and within each quadrat, collected five composite surface soil samples from the surface 10 cm soil under the dominant vegetation types (e.g., trees, shrubs, grasses etc). Within each 50x50m plot, three 50-m parallel transects were established, spaced 25 m apart. These transects were used in our vegetation surveys. The replication numbers were comparable to those in most publications within the field of knowledge.
Data collection	We collected an extensive amount of new field data from 16 soil chronosequences across global biomes (Fig. 1), and collected information for 32 topsoil, plant and microbial ecosystem properties. Detailed information about the methods used to obtain this information can be found in the Method section and in Supplementary Methods 1. Detailed information on the investigated 16 chronosequences can be found in the method section, Fig. 1 and Supplementary Table 1. We also collected data from 48 global soil chronosequences. Information on these locations can be found in the method section, Fig. 3, Supplementary Table 2 and Supplementary Methods 1 and 2.
Timing and spatial scale	Soil and vegetation data were collected using standardized protocols between 2016 and 2017 from 16 soil chronosequences located in nine countries and six continents (Fig. 1 and Supplementary Table 1)
Data exclusions	No data were excluded from analyses.
Reproducibility	Information about the sampled locations and methods used in this paper are included in our material and methods
Randomization	Field-based samples were collected, a priori, for sites of known soil age and data analysed using these a priori classes. Therefore there was no allocation of data to classes a posteriori.
Blinding	N/A

Did the study involve field work? ☒ Yes ☐ No

Field work, collection and transport

Field conditions	We conducted a field survey in 16 globally distributed soil chronosequences. These chronosequences were selected to include a wide range of climates (tropical, temperate, continental, polar and arid) and vegetation types (including grasslands, shrublands, forests, and forblands).
Location	We conducted a global field survey in 16 soil chronosequences from nine countries and six continents. Detailed information on these locations can be found in the method section, Fig. 1 and Supplementary Table 1. We also collected data from 48 global soil chronosequences. Information on these locations can be found in the method section, Fig. 3, Supplementary Table 2 and Supplementary Methods 1 and 2.
Access and import/export	Samples were collected by all authors in their respective locations and using local permits.
Disturbance	This study did not cause any environmental disturbance

Reporting for specific materials, systems and methods

We require information from authors about some types of materials, experimental systems and methods used in many studies. Here, indicate whether each material, system or method listed is relevant to your study. If you are not sure if a list item applies to your research, read the appropriate section before selecting a response.

Materials & experimental systems

n/a	Involved in the study
☒	☐ Antibodies
☒	☐ Eukaryotic cell lines
☒	☐ Palaeontology
☒	☐ Animals and other organisms
☒	☐ Human research participants
☒	☐ Clinical data

Methods

n/a	Involved in the study
☒	☐ ChIP-seq
☒	☐ Flow cytometry
☒	☐ MRI-based neuroimaging
